# Microbiome Analysis for Wastewater Surveillance during COVID-19

**DOI:** 10.1128/mbio.00591-22

**Published:** 2022-06-21

**Authors:** Kyle D. Brumfield, Menu Leddy, Moiz Usmani, Joseph A. Cotruvo, Ching-Tzone Tien, Suzanne Dorsey, Karlis Graubics, Brian Fanelli, Isaac Zhou, Nathaniel Registe, Manoj Dadlani, Malinda Wimalarante, Dilini Jinasena, Rushan Abayagunawardena, Chiran Withanachchi, Anwar Huq, Antarpreet Jutla, Rita R. Colwell

**Affiliations:** a Maryland Pathogen Research Institute, University of Maryland, College Park, Maryland, USA; b University of Maryland Institute for Advanced Computer Studies, University of Maryland, College Park, Maryland, USA; c Essential Environmental and Engineering Systems, Huntington Beach, California, USA; d Geohealth and Hydrology Laboratory, Department of Environmental Engineering Sciences, University of Florida, Gainesville, Florida, USA; e Joseph Cotruvo and Associates LLC, Washington, DC, USA; f Maryland Department of Environment, Baltimore, Maryland, USA; g CosmosID Inc., Germantown, Maryland, USA; h Inspection Experts Inc., Columbia, Maryland, USA; University of Michigan-Ann Arbor

**Keywords:** COVID-19, SARS-CoV-2, wastewater, microbiome, wastewater-based epidemiology, wastewater monitoring, wastewater surveillance, RT-qPCR, DNA sequencing, RNA sequencing, metagenomics, whole metagenome sequencing, metatranscriptomics, shotgun sequencing, risk assessment, environmental risk

## Abstract

Wastewater surveillance (WS), when coupled with advanced molecular techniques, offers near real-time monitoring of community-wide transmission of SARS-CoV-2 and allows assessing and mitigating COVID-19 outbreaks, by evaluating the total microbial assemblage in a community. Composite wastewater samples (24 h) were collected weekly from a manhole between December 2020 and November 2021 in Maryland, USA. RT-qPCR results showed concentrations of SARS-CoV-2 RNA recovered from wastewater samples reflected incidence of COVID-19 cases. When a drastic increase in COVID-19 was detected in February 2021, samples were selected for microbiome analysis (DNA metagenomics, RNA metatranscriptomics, and targeted SARS-CoV-2 sequencing). Targeted SARS-CoV-2 sequencing allowed for detection of important genetic mutations, such as spike: K417N, D614G, P681H, T716I, S982A, and D1118H, commonly associated with increased cell entry and reinfection. Microbiome analysis (DNA and RNA) provided important insight with respect to human health-related factors, including detection of pathogens and their virulence/antibiotic resistance genes. Specific microbial species comprising the wastewater microbiome correlated with incidence of SARS-CoV-2 RNA, suggesting potential association with SARS-CoV-2 infection. Climatic conditions, namely, temperature, were related to incidence of COVID-19 and detection of SARS-CoV-2 in wastewater, having been monitored as part of an environmental risk score assessment carried out in this study. In summary, the wastewater microbiome provides useful public health information, and hence, a valuable tool to proactively detect and characterize pathogenic agents circulating in a community. In effect, metagenomics of wastewater can serve as an early warning system for communicable diseases, by providing a larger source of information for health departments and public officials.

## INTRODUCTION

Global change, namely, climate variability, urbanization, rapid long-distance travel, and the projected human population growth, has increased the risk of infectious disease outbreaks (1). Pathogen cross-over from animal reservoirs into human populations increasingly has been reported to occur, both in frequency and diversity, over the last century, namely, Ebola, HIV/AIDS, West Nile Virus, and Middle East respiratory syndrome. The United Nations predicts further emergence of zoonotic diseases as habitats are ravaged by wildlife exploration, unsustainable farming practices, and climate variability (2).

In December 2019, several cases of an unknown pneumonia were reported in the Hebei province of Central China, marking the beginning of the global COVID-19 pandemic caused by Severe Acute Respiratory Syndrome Coronavirus 2 (SARS-CoV-2), and declared by the World Health Organization in March 2020 (3). By January 2022, over 400 million cases and more than 5 million deaths were reported (4). A significant finding of the COVID-19 pandemic to date has established aerosols as the primary transmission route of SARS-CoV-2 (5). However, the role of the ambient and built environment, as well as climate and weather processes, have begun to be considered important factors in promoting transmission, namely, via aerosolized viruses (6).

Progression of the pandemic has been monitored primarily by counting the number of individuals testing positive for presence of SARS-CoV-2, prompted mainly by onset of symptoms of the disease, generally appearing 2 to 4 days after exposure but, in some cases, taking up to 2 weeks to present after infection (7). However, in a large proportion of transmission events, presymptomatic (8) and even asymptomatic (9) transmission has been observed. Clinical testing programs provide information only on a subset of individuals that consent to testing (10). Over the counter self-tests, namely, antigenic tests which are considered less accurate than quantitative reverse transcriptase PCR (RT-qPCR), especially for asymptomatic subjects (11), have become increasingly popular but rely on individuals correctly performing the test and reporting positive results to health departments (12). Hence, the use of clinical data for estimations of COVID-19 prevalence has the potential to be biased, based on factors such as health-seeking behavior, undertesting of asymptomatic cases, inequitable access to testing, and selective testing mandates applying only to certain groups or regions (10).

Clinical diagnosis of COVID-19 is typically determined by detection of acute infection targets in the genome of SARS-CoV-2 present in nasopharyngeal, nasal, and saliva swab samples (13, 14). However, the virus can also be detected in specimens from other sites of infected individuals, notably in feces (15, 16). Proposed as a complement to clinical testing, wastewater surveillance (WS; also known as wastewater monitoring or wastewater-based epidemiology) of samples collected from treatment systems of communities has been used for early detection of community-wide disease prevalence, notably for poliovirus (17–19), noroviruses (20), flu (21), and recently COVID-19 (22). WS has also been employed to assess diverse factors influencing communitywide health, such as monitoring consumption of local diets, alcohol, illicit drugs, and tobacco, and evaluating exposure to hazardous chemicals and pharmaceuticals (23, 24). WS of confined populations, e.g., inhabitants of a single building (25) or university dormitory (26) or small community sewage collection systems (SCS) of a neighborhood (27), can be considered less biased, because the evaluation is pooled contributions of all individuals served by a given catchment area, compared with clinical testing, where only a minority of consenting individuals are tested routinely. Therefore, WS can be a useful public health tool to proactively study emergence and spread of COVID-19 (28). However, it is worth noting that WS, while able to monitor a given community, is most useful in municipalities with SCS infrastructure, and representation of rural towns may be diminished given that WS has the potential to miss those households served by septic systems.

RT-qPCR is widely recognized as the “gold standard” for detection of SARS-CoV-2 RNA in wastewater samples around the world (29–35). However, detection and enumeration only of SARS-CoV-2 markers does not provide comprehensive information, namely, the full range of enteric microorganisms and virulence and antimicrobial resistance (AMR) associated genes present in a community at a given time. Shotgun metagenomic sequencing (SMS) offers an effective WS tool (36–38) that allows bacterial, archaeal, viral, fungal, and protozoan microbiome community members to be identified to subspecies taxonomic level and characterized (39). While both RT-qPCR and SMS are established methods, the interaction of SARS-CoV-2 with other members of the microbial community, both in the patient and environment, remain understudied.

**FIG 1:**
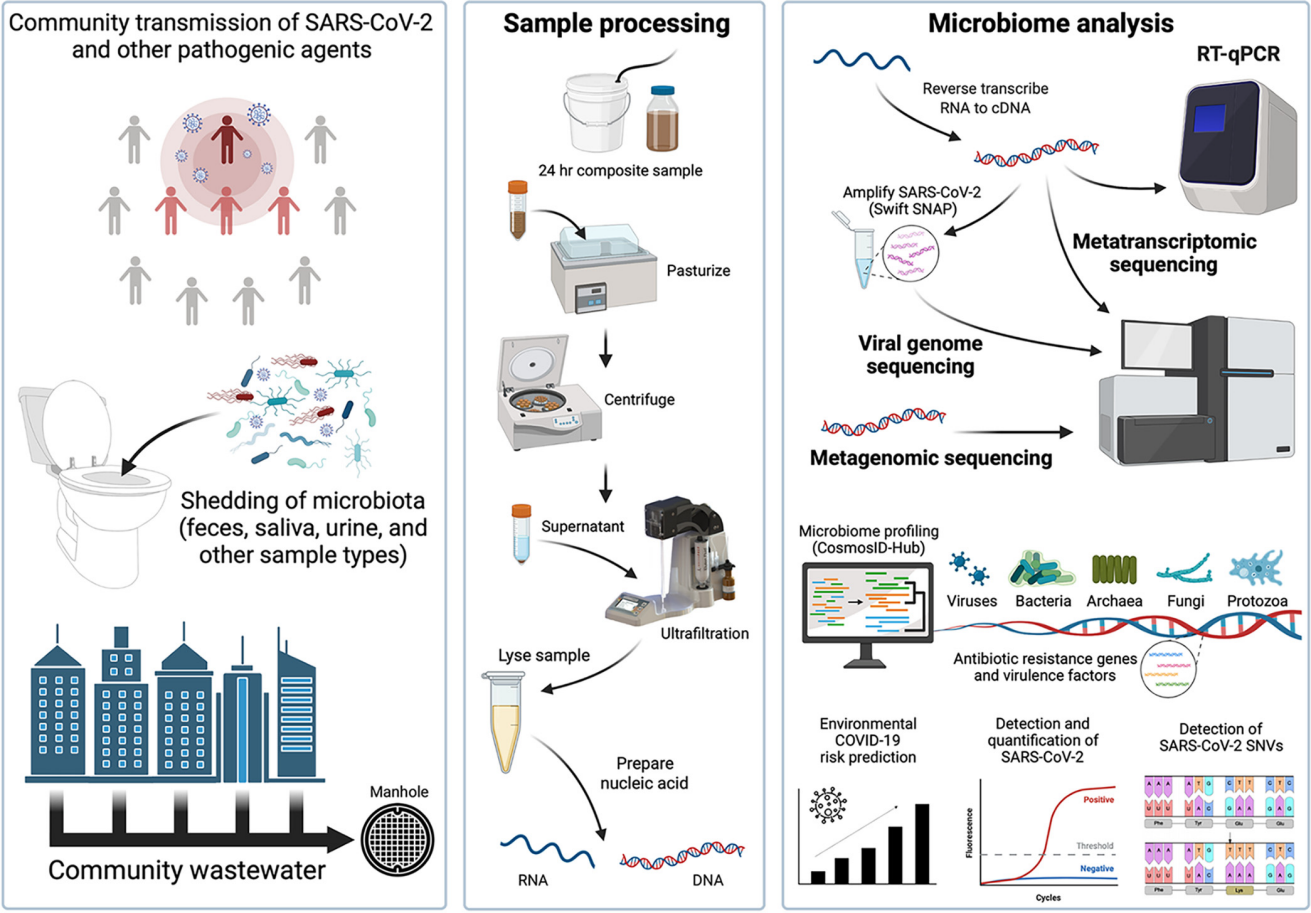
Schematic representation of sample processing. Image created using BioRender.

This investigation is the first to employ a complete molecular toolset, i.e., DNA metagenomics, RNA metatranscriptomics, targeted amplicon sequencing, and RT-qPCR, along with advanced bioinformatics, to analysis of community wastewater (Fig. 1). We demonstrated the feasibility and application of assessing trends of SARS-CoV-2 RNA in wastewater samples collected from a SCS in Maryland, USA, for approximately 1 year, from December 2020 to November 2021, and simultaneously profiled the microbiome component (DNA and RNA; bacteria, archaea, fungi, protists, and viruses, and presence of virulence and AMR associated genes) of a subset of samples. We showed significant positive correlation between environmental risk scores and prevalence of COVID-19 cases in the study area. Results suggest remote sensing can be used in near real-time to identify geographic regions with increased COVID-19 risk scores, and the wastewater microbiome is a useful public health tool to detect and characterize pathogenic agents circulating in communities, hence, an early warning system for communicable diseases.

## RESULTS

### SARS-CoV-2 RT-qPCR.

####  (i) RT-qPCR quality controls.

Calibration model performance parameters, including slope and y-intercept parameters, linearity (*R^2^*), and Amplification efficiency (AE), are provided in the supporting information (Data set S2). Calibration model *R^2^* values for Nucleocapsid Phosphoprotein (N protein) were ≥ 0.98 and *E*-values ranged from 0.73 to 1.02 (average, 0.91). All no template controls (NTCs) were negative for detection of SARS-CoV-2 RNA. Based on internal control (IC) MS2 bacteriophage testing, amplification inhibition was not observed. Hence, all RNA extracts included in subsequent analysis exhibited negligible matrix interference and passed sample processing control.

10.1128/mbio.00591-22.8 DATA SET S2 Detection and quantification of SAR-CoV-2 via qRT-PCR. Download Data Set S2, XLSX file, 0.01 MB. Copyright © 2022 Brumfield et al. 2022 Brumfield et al. https://creativecommons.org/licenses/by/4.0/ This content is distributed under the terms of the Creative Commons Attribution 4.0 International license.

#### (ii) Detection of SARS-CoV-2 RNA and association with epidemiological data.

Detection of genetic markers against SARS-CoV-2 N protein was determined from wastewater concentrates, representing temporal sampling between December 30, 2020 and November 16, 2021. Of the 48 measurements, 24 (50%) were negative for detection of N protein, 23 were within the range of quantification (ROQ), and one (11/2/2021; 6.53 × 10^6^ N copies/L) was above the ROQ. Excluding the measurement above ROQ, N protein was detected at the highest concentration during the week of February 17, 2021 (7.80 × 10^5^ N copies/L), followed by October 5, 2021 (1.65 × 10^5^ N copies/L) and at varying concentrations throughout the study period (Data set S2).

**FIG 2:**
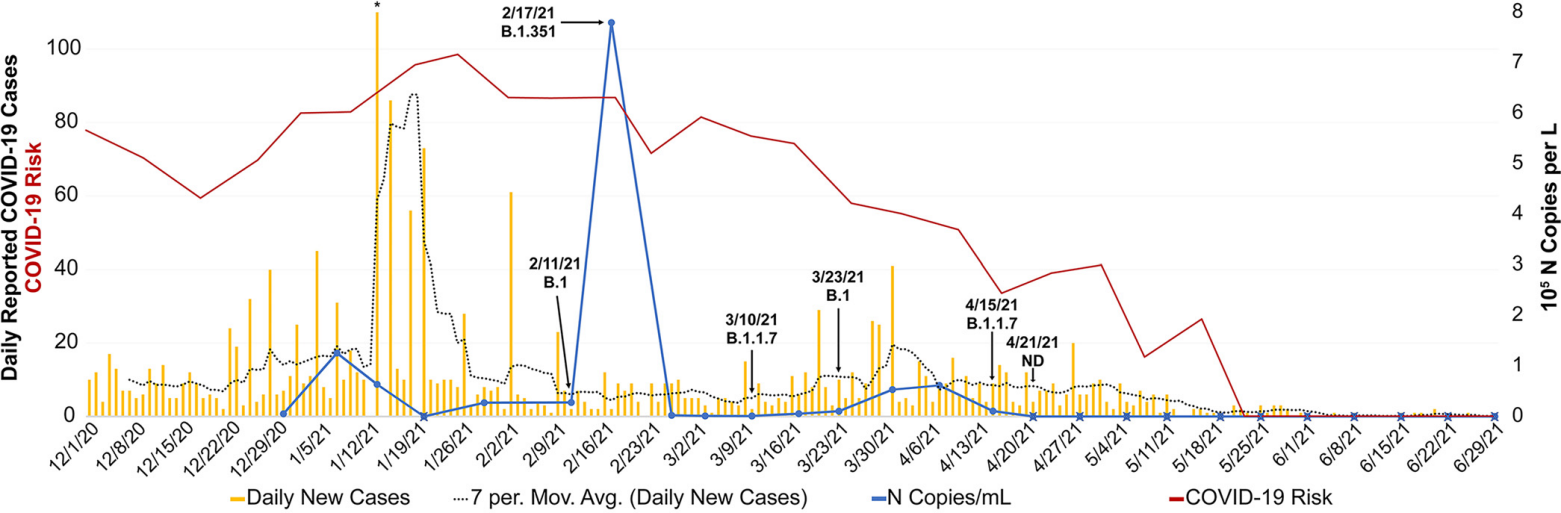
Reported COVID-19 cases and associated risk (left axis) and detection of SARS-CoV-2 N protein via RT-qPCR (right axis). Total daily number of reported COVID-19 cases for the ZIP code where the study took place was retrieved from the Maryland Department of Health (81). Asterisks represent truncated bar plot as 273 cases were reported on January 13, 2021. COVID-19 environmental predictive risk was calculated using ambient air temperature and dew point, as described previously (6). The risk score, a ratio between 0 and 1, is normalized on a scale of 0 to 100, with 100 being the highest risk of transmission. Wastewater samples were collected between December 30, 2020 and June 29, 2021. Concentration of SARS-CoV-2 was determined by RT-qPCR analysis. Blue circle indicates detection of SARS-CoV-2; blue circle with superimposed “X” indicates SARS-CoV-2 was not detected. Arrows indicate date of samples included for advanced molecular analysis (DNA metagenomics, RNA metatranscriptomics, and targeted RNA sequencing). SNVs commonly associated with known variations detected in consensus sequences are shown as SARS-CoV-2 Pango lineages. Gold bars, daily new cases; dotted black line, 7-day moving average of daily new cases; blue line, N copies/L; red line, COVID-19 risk; ND, not detected.

For the location where the study was conducted, the number of reported COVID-19 cases was higher during the winter compared with warmer months, with a spike in number of COVID-19 cases reported during the second week of January 2021. Generally, the amount of SARS-CoV-2 RNA detected in the community wastewater showed a similar pattern to the number of reported COVID-19 cases (Fig. 2). However, because the population served by this SCS represents *ca.* 3% of the total population included in the epidemiological reports, and viral shedding in feces of infected individuals is extremely variable, no statistical comparisons between the number of COVID-19 cases and concentrations of SARS-CoV-2 RNA were determined.

#### (iii) COVID-19 environmental predictive risk.

Previous work showed a tolerable ambient temperature range of 17°C to 24°C corresponded to a decrease in number of COVID-19 cases in the human population (6). A schematic representation of this generalizable hypothesis governing the dynamics of COVID-19 disease in a human population, where AT and DPT play a role in potential aerosolization of SARS-CoV-2 via particles in ambient and built environments (indoors) is shown in the supporting information (Fig. S1). During this investigation, the change in COVID-19 cases and detected concentration of SARS-CoV-2 RNA in wastewater followed a similar pattern to the change in the environmental risk scores (Fig. 2). Using parametric (Pearson) and nonparametric (Kendall tau) measures to determine the association between predicative risk scores and reported cases yielded statistically significant (*P* < 0.01) positive correlation of 0.58 and 0.54, respectively. On a weekly scale between December 2020 and November 2021 (47 weeks), when the risk score was greater than 0.5, SARS-CoV-2 was detected in wastewater samples 88% of the time (15/18 weeks), whereas when the risk score was below 0.5, SARS-CoV-2 was detected at low concentration 31% of the time (9/29 weeks). The greatest risk scores were detected during the end of January and the beginning of February, which coincided with highest number of COVID-19 cases. During the week of April 19, COVID-19 risk scores dropped below 0.5 and remained low throughout the summer, with slight increases in risk occurring in July and August. During this period of time only a few COVID-19 cases were reported and SARS-CoV-2 RNA was not detected by RT-qPCR in the wastewater samples. Subsequently, environmental risk scores increased during the fall, reaching above 0.5 in November 2021, which was mirrored by both increases in the number of COVID-19 cases and detection of SARS-CoV-2 at higher concentrations in wastewater samples. However, it is worth noting that various social and demographic factors, e.g., asymptomatic carriers, unreported home tests, human behavior, etc., impact the number of reported cases and could not be controlled for in this study. Therefore, we present calculated environmental COVID-19 risk scores as a ratio (Data set S3).

10.1128/mbio.00591-22.2 FIG S1 Hypothesis for environmental COVID-19 risk prediction, adapted from Usmani et al. (Am J Trop Med Hyg 106:1-9, 2020, https://doi.org/10.4269/ajtmh.21-0328). Download FIG S1, DOCX file, 0.4 MB. Copyright © 2022 Brumfield et al. 2022 Brumfield et al. https://creativecommons.org/licenses/by/4.0/ This content is distributed under the terms of the Creative Commons Attribution 4.0 International license.

10.1128/mbio.00591-22.9 DATA SET S3 Calculated environmental COVID-19 risk scores. Download Data Set S3, XLSX file, 0.01 MB. Copyright © 2022 Brumfield et al. 2022 Brumfield et al. https://creativecommons.org/licenses/by/4.0/ This content is distributed under the terms of the Creative Commons Attribution 4.0 International license.

#### (iv) Characteristics of SARS-CoV-2 variants.

**FIG 3:**
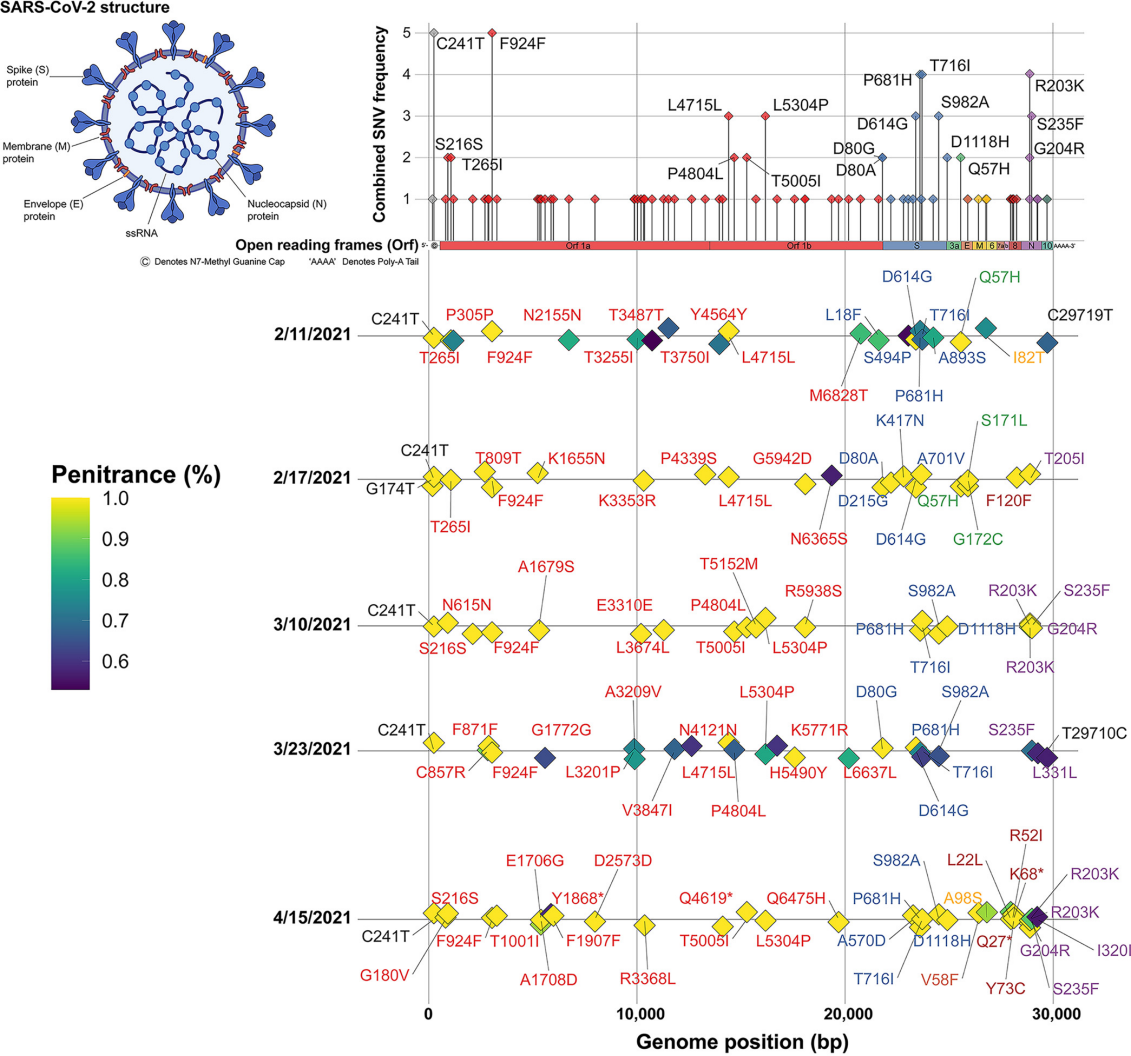
Penetrance of SARS-CoV-2 variants recovered from wastewater. Bar plot (top panel) showing the combined number of samples for which each genetic variant was detected. Heatmap (bottom panel) showing the penetrance (%) of reads associated with each genetic variant detected in SARS-CoV-2 consensus sequences recovered from wastewater relative to the total number of reads mapping to a given position. Inset shows SARS-CoV-2 structure.

Using a targeted sequencing approach, presence of SARS-CoV-2 was confirmed, and consensus sequences were characterized (Fig. 3). Presence of SARS-CoV-2 was confirmed in five of the six samples processed for targeted sequencing. The sample collected on April 21, 2021 was negative for presence of SARS-CoV-2 RNA, in agreement with results of the RT-qPCR analysis for these samples. An increase in the concentration of SARS-CoV-2 RNA in wastewater was observed on February 17, and genetic variations detected in the consensus sequence of this sample were similar to those associated with B.1.351 (Beta, V2). Prior to the February increase, various mutations commonly described in basal lineages were detected, and genetic variations associated with the B.1.1.7 (Alpha, V1) lineage were detected in consensus sequences recovered in April. Characteristics of SARS-CoV-2 variants recovered from wastewater, including the percent of reads that contained each mutation, are shown in Fig. 3. Across consensus sequences recovered from wastewater, between 20 (February 11, 2021) and 32 (April 15, 2021) single nucleotide variants (SNVs) were detected. All samples encoded a pyrimidine nucleotide variation at position 241 in the 5′-untranslated region of the virus genome (C241T). The synonymous C3037T (ORF1a:F924F) mutation was also detected at all time points. Two mutations were observed at genome position 21,801, including S:D80A (February 17, 2021) and S:D80G (March 10, 2021). Nonsynonymous mutations were detected frequently in the structural protein coding regions of spike (D614G, P681H, T716I, and S982A) and nucleocapsid (R203K, G204R, and S235F). However, SNVs in the envelope (E) and membrane (M) proteins were less common. The E:V57F mutation was observed in the sample collected on April 15, 2021, but no other E protein SNVs were detected. Detected membrane SNVs include I81T detected in the sample collected on February 11, 2021 and A97S detected on April 15, 2021. A genomic deletion was observed on March 10, 2021 (ORF1ab: GTCTGGTTTT11287G; penetrance = 100%), and three stop codons with 100% penetrance (ORF1ab:Q4618*, ORF8:Q26*, and OFR8:K67*) were detected in the sample collected on April 15, 2021. Other notable mutations detected in more than one sample include two synonymous mutations in ORF1ab (S216S and L4715L), four nonsynonymous mutations detected in ORF1ab (T265I, 4804L, T5005I, L5304P) and one nonsynonymous SNV in ORF3a (Q57H).

### Metagenomic data analysis.

#### (i) Community microbiome.

**FIG 4:**
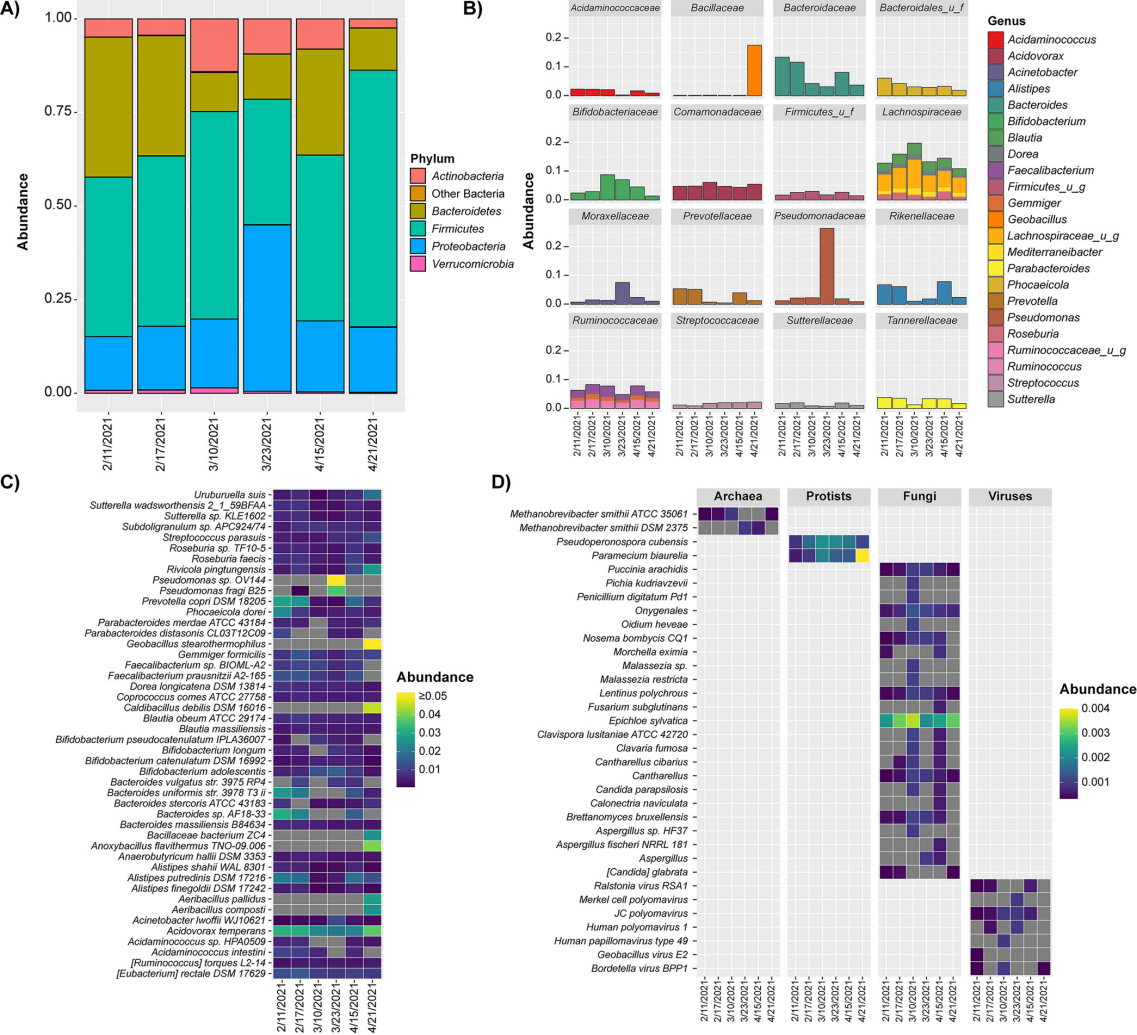
Microbiome profiles employing DNA metagenomic sequencing. (A) Stacked bar plot showing relative abundance of detected bacterial phyla. Top five most abundant phyla are shown, and all other phyla are labeled “other bacteria.” (B) Stacked bar plots showing relative abundance of detected bacterial genera. Shown are the 20 most abundant genera grouped by family. (C) Heatmap showing universal kingdom relative abundance of most abundant species. Shown are most abundant taxa, representing >0.2% relative abundance. (D) Heatmap showing universal kingdom relative abundance of detected archaea, protists, fungi, and viruses.

Shotgun metagenomics and metatranscriptomics using DNA and RNA prepared from the wastewater samples generated approximately 3.92 × 10^8^ and 5.53 × 10^8^ reads across the raw sequence libraries, with a mean of 65.3 and 92.1 million unique reads for metagenomic and metatranscriptomic samples, respectively (Data set S4). Alpha diversity was calculated using CHAO1 index and ranged from 1,059 to 1,358 (average = 1,180.5) for total bacteria and from 29 to 51 (average = 43.3) for total RNA viruses (Table S1). Bacterial communities in wastewater samples were analyzed by three-dimensional PCoA using Bray-Curtis dissimilarity index (Fig. S2A), where distance between points indicates degree of difference in bacterial DNA sequence composition. Each sample contained a relatively distinct bacterial composition. However, samples collected during February 2021, clustered more closely compared with those collected during March and early April. Interestingly, the April 21, 2021 sample that tested negative for SARS-CoV-2 RNA by RT-qPCR did not cluster with any of the other samples. Bacteria, archaea, fungi, protozoa, and viruses identified by DNA metagenomics are shown in Fig. 4, representing relative abundance (RA) of microbial species.

10.1128/mbio.00591-22.3 FIG S2 Diversity of bacterial communities profiled in wastewater with respect to SARS-CoV-2. (A) Principal coordinate analysis of bacterial communities. Water samples were categorized into clusters by PCoA using the Bray-Curtis distance metric based on relative abundance of bacterial species. Distance between points indicates degree of dissimilarity in bacterial composition, ranging from zero (samples share the same species abundances) to one (samples contain completely different species abundances). The percent variation explained by each axis is indicated. (B) Venn diagram representing bacterial communities. The number of shared and exclusive bacteria are shown relative to detection genetic mutations associated with SARS-CoV-2 variants of concern, i.e., Alpha (V1) and Beta (V2), along with a sample negative for detection of SARS-CoV-2 (ND). Download FIG S2, DOCX file, 0.4 MB. Copyright © 2022 Brumfield et al. 2022 Brumfield et al. https://creativecommons.org/licenses/by/4.0/ This content is distributed under the terms of the Creative Commons Attribution 4.0 International license.

10.1128/mbio.00591-22.6 TABLE S1 Alpha diversity indices of detected bacteria (DNA) and viruses (RNA). Download Table S1, DOCX file, 0.02 MB. Copyright © 2022 Brumfield et al. 2022 Brumfield et al. https://creativecommons.org/licenses/by/4.0/ This content is distributed under the terms of the Creative Commons Attribution 4.0 International license.

10.1128/mbio.00591-22.10 DATA SET S4 Sequencing statistics and accession numbers. Download Data Set S4, XLSX file, 0.01 MB. Copyright © 2022 Brumfield et al. 2022 Brumfield et al. https://creativecommons.org/licenses/by/4.0/ This content is distributed under the terms of the Creative Commons Attribution 4.0 International license.

For all wastewater samples, significant differences were observed in the microbiota (Fig. 4, 5A). While SARS-CoV-2 RNA was detected in five of six samples by RT-qPCR, the virus was not detected by RNA metatranscriptomics in any of the samples. However, there appeared to be a relation between the number of shared and exclusive bacteria, i.e., unique taxa detected in a set of samples and not detected in other samples, and the lineage of SARS-CoV-2 that was detected. A Venn diagram representing bacteria profiles, with respect to detection of genetic mutations commonly associated with SARS-CoV-2 variants of concern, is included in the supporting information (Fig. S2B). An increased number of exclusive bacteria was observed in samples corresponding to detection of Alpha variants (486 spp.) compared with samples where Beta variants were detected or samples where SARS-CoV-2 was not detected, which contained 137 and 127 exclusive species, respectively. The sample negative for SARS-CoV-2, collected on April 21, 2021, contained fewer shared bacteria with samples where Beta variants were detected (26 spp.) compared with samples where Alpha variants were detected (127 spp.). A total of 167 shared species was observed between samples with Alpha variants and samples with Beta variants. Analysis of the RNA viral community showed the sample collected on March 23 contained the greatest number of exclusive taxa (18), while the other samples contained relatively few exclusive taxa (≤5).

**FIG 5:**
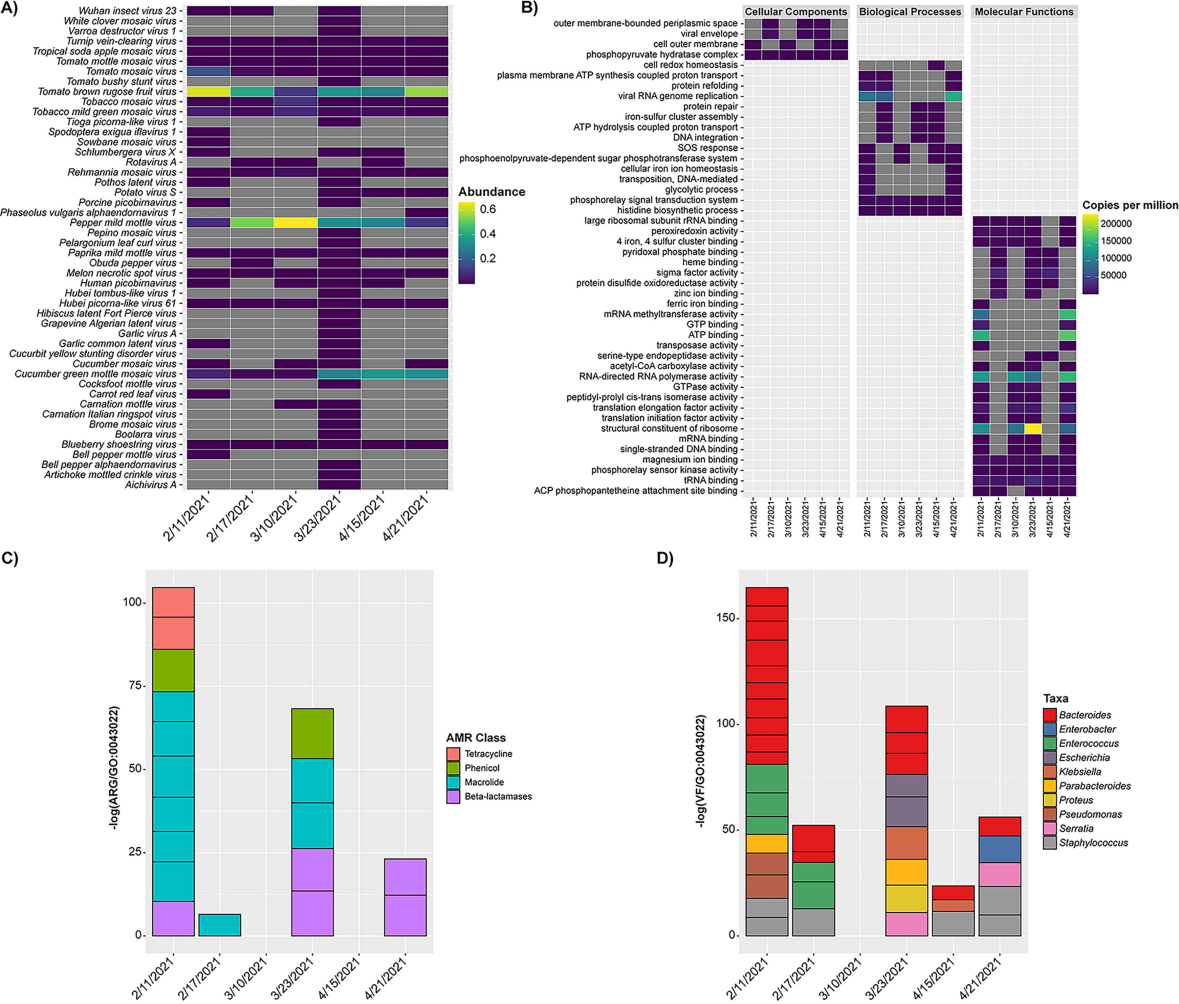
Microbiome profiles employing RNA metatranscriptomic sequencing. (A) Heatmap showing relative abundance of detected RNA viruses. (B) Stacked bar plot showing expression of detected AMR genes. Shown are individual AMR genes colored by class. Qualitative expression is shown relative to GO:0043022. (C) Stacked bar plot showing expression of detected virulence factors. Shown are individual virulence-associated genes colored by taxa. Qualitative expression is shown relative to GO:0043022. (D) Heatmap showing relative expression of detected gene ontology terms. Shown are relative expression (copies per million reads) of the most abundant gene ontology terms grouped by category.

A core microbiome, i.e., taxa detected in all samples, was observed for all of the wastewater samples, with 544 bacterial species detected by DNA metagenomics and 14 RNA viruses detected by metatranscriptomics. RNA metatranscriptomics also allows for detection of active members of the microbiome. Overall, a similar core microbiome was detected between RNA and DNA sequencing. Microbiome profiles employing RNA metatranscriptomics, including the most abundant genera detected by both sequencing methods is included in the supporting information (Fig. S3). Generally, core bacteria detected were assigned to phyla *Firmicutes*, *Proteobacteria*, *Bacteroidetes*, or *Actinobacteria*. At the family level, *Lachnospiraceae*, *Bacteroidaceae*, and *Ruminoccoccaceae* were the most abundant core bacteria detected. However, there was a substantial increase in *Pseudomonadaceae*, namely, members of the genus Pseudomonas, detected in the March 23, 2021 sample (>20% RA), and *Bacillaceae*, primarily members of the genus *Geobacillus*, detected in the sample negative for SARS-CoV-2 (April 21; >15% RA). Across all samples, the most abundant core microbial species were Acidovorax temperans (bacteria; *ca.* 3 to 4% RA) and *Epichloe sylvatica* (fungi; *ca.* 0.02 to 0.04% RA). Pepper mild mottle virus and tomato brown rugose fruit virus were the most abundant RNA viruses detected in the core wastewater microbiome. Notable exclusive microbial species detected in the sample negative for SARS-CoV-2 (April 21) include Geobacillus stearothermophilus, Caldibacillus debilis, and Anoxybacillus flavithermus. With respect to samples positive for detection of SARS-CoV-2 RNA, the results showed a minimal number of exclusive microbial species detected in samples at high SARS-CoV-2 concentrations compared with lower concentrations.

10.1128/mbio.00591-22.4 FIG S3 Microbiome profiles employing RNA metatranscriptomic sequencing. (A) Stacked bar plot showing relative abundance of detected bacterial phyla. (B) Stacked bar plots showing relative abundance of detected bacterial genera. Shown are the most abundant genera, grouped by family, detected by both DNA and RNA shotgun sequencing. Download FIG S3, DOCX file, 0.2 MB. Copyright © 2022 Brumfield et al. 2022 Brumfield et al. https://creativecommons.org/licenses/by/4.0/ This content is distributed under the terms of the Creative Commons Attribution 4.0 International license.

#### (ii) Pathogenic wastewater-associated microorganisms.

Incorporating an *in situ* control for DNA metagenomics allowed for absolute cell number quantification of detected microbiota following metagenomic profiling (Fig. S4). The sample collected on February 17, 2021 contained the highest concentration of microbial cells/L (1.34 × 10^10^), followed by April 21 (1.00 × 10^10^) and February 11 (8.42 × 10^9^). The lowest concentration was detected on March 10, 2021 (1.23 × 109 cell/L). Most microbial cells were profiled as bacteria (min = 1.21 × 10^9^, March 10; max = 1.33 × 10^10^, February 17; average = 6.30 × 10^9^), followed by fungi (min = 5.39 × 10^6^, March 23; max = 5.97 × 10^7^, February 17; average = 2.64 × 10^7^) and protists (min = 5.39 × 10^6^, March 23; max = 5.47 × 10^7^, April 21; average = 1.89 × 10^7^). DNA viruses were detected in all samples at low abundance (<2 × 105 cells/L), and archaea were rarely detected (≤1 × 103 cells/L). Table 1 shows the number of multiple pathogenic microorganisms detected in wastewater samples. Members of the *Enterobacteriaceae* family, namely, Escherichia coli and Klebsiella pneumoniae, and were detected in all samples. *Aeromonas hydrophilia* was also detected in all samples, but at lower cell number. Vibrio cholerae was detected at a concentration of 1.16 × 106 cells/L in the February 11 sample, and at a lower cell number on February 17 and March 23. Human polyomavirus 2 was the most common DNA virus, detected in all samples except the sample collected on April 21.

**TABLE 1:** Quantification of microorganisms employing DNA metagenomics^*a*^

Genus	Species	Illness/description	2/11/21	2/17/21	3/10/21	3/23/21	4/15/21	4/21/21
Bacteria								
*Aeromonas*	*A. hydrophilia*	Gastroenteritis; septicemia	4.22 × 10^5^	6.93 × 10^5^	2.5 × 10^4^	3.3 × 10^4^	5.2 × 10^4^	3.68 × 10^5^
*Bacteroides*	B. thetaiotaomicron	FIB	5.21 × 10^7^	6.33 × 10^7^	2.04 × 10^6^	2.54 × 10^6^	1.44 × 10^7^	5.88 × 10^6^
*Bifidobacterium*	B. adolescentis	FIB	5.29 × 10^7^	1.02 × 10^8^	2.89 × 10^7^	4.83 × 10^7^	4.33 × 10^7^	3.6 × 10^7^
	*B. dentium*	FIB	1.16 × 10^6^	6.93 × 10^5^	2.24 × 10^5^	7.91 × 10^5^	1.19 × 10^6^	1.23 × 10^5^
*Burkholderia*	*B. vietnamiensis*	Respiratory illness	1.05 × 10^5^	-	-	-	5.2 × 10^4^	-
Campylobacter	Campylobacter *spp.*^*b*^	Campylobacteriosis	1.05 × 10^5^	1.73 × 10^5^	-	-	-	-
Escherichia	E. coli	FIB	1.72 × 10^9^	1.01 × 10^9^	1.66 × 10^6^	2.18 × 10^6^	2.79 × 10^6^	8.09 × 10^6^
*Enterococcus*	*E. avium*	FIB	2.64 × 10^6^	3.47 × 10^5^	-	-	-	1.23 × 10^5^
	*E. casseliflavus*	FIB	2.11 × 10^5^	-	-	6.6 × 10^4^	-	-
	E. faecalis	FIB	9.49 × 10^5^	-	1.74 × 10^5^	5.6 × 10^5^	-	-
	E. faecium	FIB	1.37 × 10^6^	6.93 × 10^5^	2.48 × 10^5^	4.81 × 10^6^	3.1 × 10^5^	1.23 × 10^5^
Klebsiella	K. pneumoniae	Pneumonia; UTI	6.38 × 10^6^	9.27 × 10^6^	1.78 × 10^6^	7.35 × 10^6^	4.31 × 10^6^	4.44 × 10^6^
*Legionella*	*L. septentrionalis*	Respiratory illness	-	-	-	6.6 × 10^4^	5.2 × 10^4^	-
*Methanobrevibacter*	M. Smithii	FIB	7.38 × 10^5^	2.08 × 10^6^	1.74 × 10^5^	8.24 × 10^5^	1.40 × 10^6^	1.35 × 10^6^
Mycobacterium	Nontuberculosis *Mycobacterium*^*c*^	Respiratory illness; skin infections	-	-	-	-	-	1.23 × 10^5^
Salmonella	S. enterica	Gastroenteritis	5.27 × 10^5^	3.47 × 10^5^	-	-	5.2 × 10^4^	1.23 × 10^5^
*Shigella*	S. dysenteriae	Dysentery	1.05 × 10^5^	-	-	-	-	-
*Vibrio*	V. cholerae	Cholera	1.16 × 10^6^	1.73 × 10^5^	-	3.3 × 10^4^	-	-
Viruses								
*Papillomaviridae*	Human *papillomavirus*	HPV	-	-	4 × 10^3^	-	-	-
*Polyomaviridae*	Human polyomavirus 2^*d*^	Progressive multifocal leukoencephalopathy	1.06 × 10^5^	1.34 × 10^5^	2.1 × 10^4^	2 × 10^4^	5.2 × 10^4^	-
	Merkel cell polyomavirus	Merkel cell carcinoma	-	-	-	3 × 10^3^	-	-
Fungi								
*Malassenzia*	*M. restricta*	Skin infection	-	-	2.34 × 10^5^	-	-	-
*Candida*	*Candida spp.* ^*e*^	Candidiasis	1.97 × 10^5^	2.48 × 10^5^	4.85 × 10^5^	-	1.40 × 10^5^	2.01 × 10^5^
Aspergillus	Aspergillus *spp.*^*f*^	Lung infection	-	-	4.6 × 10^4^	-	1.25 × 10^5^	-

a Number of each taxon was normalized to cell number of an *in situ* control (ZymoBIOMICS High Microbial Load Spike-in Control I; Zymo Research, Irvine, CA, USA) comprised of Imtechella halotolerans (Gram-negative) and Allobacillus halotolerans (Gram-positive). Quantification of microbiota is shown as cells/L. UTI, Urinary tract infection; FIB, fecal indicator bacterium; HPV, Human *papillomavirus*; -, not detected.

b Campylobacter gracilis, Campylobacter upsaliensis, Campylobacter *ureolticus*.

c Mycobacterium avium, Mycobacterium *pseudokansasii*.

d JC polyomavirus.

e Clavispora lusitaniae, Candida parapsilosis, Candida glabrata.

f Aspergillus fischeri, Aspergillus *spp*. HF37.

10.1128/mbio.00591-22.5 FIG S4 Stacked bar plots showing quantification (cell number/mL) of detected microbiota (DNA). Microbial cell number of each taxon was normalized to the cell number of an *in situ* positive control comprised of Imtechella halotolerans (Gram-negative) and Allobacillus halotolerans (Gram-positive). Quantification of microbiota are shown as cells per mL and are grouped by kingdom. Download FIG S4, DOCX file, 0.2 MB. Copyright © 2022 Brumfield et al. 2022 Brumfield et al. https://creativecommons.org/licenses/by/4.0/ This content is distributed under the terms of the Creative Commons Attribution 4.0 International license.

#### (iii) Functional analysis of wastewater microbiome.

RNA metatranscriptomics allows biological insight into gene expression and functional analysis. Fig. 5B shows the most abundant gene ontology (GO) terms profiled in each sample. Genes associated with cellular components (GO:0000015, phosphopyruvate hydratase complex), biological processes (GO:0000160, phosphorelay signal transduction system, and GO:0070180, large ribosomal subunit rRNA binding), and molecular functions (GO:0000287, magnesium ion binding, GO:0000155, phosphorelay sensor kinase activity, and GO:0000049, tRNA binding) were detected in all samples. Genes associated with viral RNA genome replication (GO:0019079) were detected in three wastewater samples (February 11 and 17, and April 21).

#### (iv) Community resistome and virulome.

AMR genes detected in the wastewater samples are shown in Fig. 5C. The largest number of AMR genes was detected in the February 11, 2021 sample (*n* = 10), followed by March 23, 2021 (*n* = 5). AMR genes associated with macrolide and beta-lactamase antibiotic classes were dominant in these samples. During the time when an increase in SARS-CoV-2 RNA was detected (February 17, 2021), macrolide was the only antibiotic class detected, at low abundance, while beta-lactamase was the only AMR class detected in the sample negative for SARS-CoV-2 RNA (April 21, 2021). A similar pattern was observed for total number of virulence associated genes (Fig. 5D). Highest frequencies of occurrence of virulence factors were detected in samples collected on February 11, 2021 (*n* = 18) and March 23, 2021 (*n* = 9). Virulence factors (VF) associated with *Bacteroides* were dominant in these samples. VF associated with Staphylococcus were detected in four of the six samples. No VF or AMR associated genes were detected in the March 10, 2021 sample, collected the week following increase in detected SARS-CoV-2 RNA on February 17, 2021.

#### (v) Co-occurrence of SARS-CoV-2 and detected microbiota.

**FIG 6:**
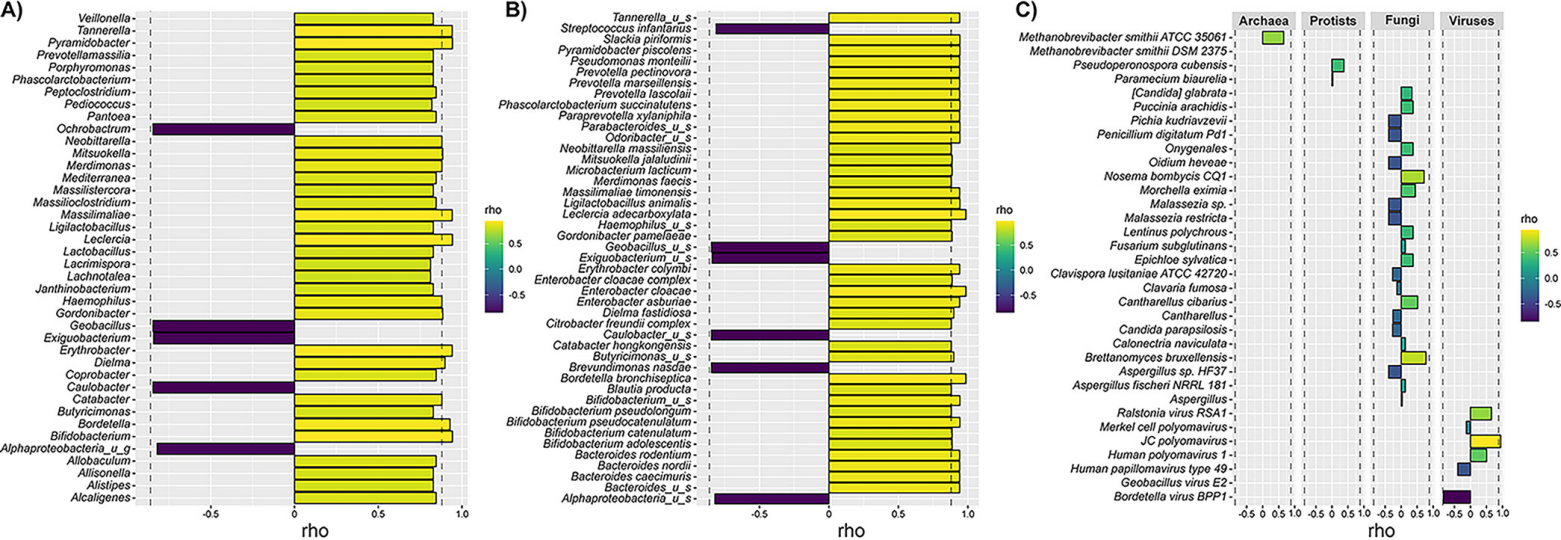
Co-occurrence of SARS-CoV-2 and microbiota. Shown are rho values, following pairwise Spearman-rank co-occurrence analysis, between concentrations of SARS-CoV-2 RNA (N copies/L) and detected microbiota (cells/L). Dotted line indicates upper and lower cutoff, for which rho values were determined by calculating pairwise Spearman rank co-occurrence for each variable with 1,000 permutation iterations. (A) Co-occurrence of SARS-CoV-2 and bacterial genera. Shown are bacterial genera with rho values (< −0.8, >0.8). (B) Co-occurrence of SARS-CoV-2 and bacterial species. Shown are bacterial species with rho values (< −0.8, >0.85). (C) Co-occurrence of SARS-CoV-2 and archaea, protists, fungi, and DNA viruses.

Normalizing DNA sequencing reads to the *in situ* control allowed successful conversion of sequencing relative abundance to cells per L, a comparable unit of conversion to concentration of SARS-CoV-2 RNA detected in wastewater following RT-qPCR. After calculating pairwise Spearman rank co-occurrence for each variable with 1,000 permutation iterations, upper and lower quantiles for permuted rho values between concentration of SARS-CoV-2 (N copies per L) and detected microbiota (cells per L) were calculated, including bacterial genera (−0.87, 0.88), species (−0.86, 0.88), and combined kingdom (−0.86, 0.88). Microbiota with highest co-occurrence correlation is shown in Fig. 6. Both positive and negative correlations were observed. However, no microbiota associated with negative correlation were significant. Of the 345 genera detected across samples, 14 were associated with positive correlation of SARS-CoV-2 RNA and greatest correlation was associated with *Bifidobacterium*, *Leclercia*, *Pyramidobacter*, *Tannerella*, *Massilimaliae*, and *Erythrobacter*. A total of 39 bacterial species were associated with significant positive correlations, most notably, Bordetella bronchiseptica, Enterobacter cloacae, and Leclercia adecarboxylata. However, various *Bifidobacterium spp*., Pseudomonas *spp., Bacteroides spp*., and *Prevotella spp*., also showed significant positive correlations. As a result of combined kingdom analysis, JC polyomavirus was the only taxa to show correlation with SARS-CoV-2, and correlations with archaea, protists, fungi, and other DNA viruses were not determined to be significant.

## DISCUSSION

### Wastewater-based epidemiology and COVID-19.

An outbreak traditionally is monitored by testing individuals presenting symptoms of the disease. However, disease incidence thus determined can be delayed or underreported because epidemiological reports rely on infected people seeking medical aid and medical practitioners reporting test results. The Centers for Disease Control and Prevention (CDC) reported *ca.* 41% of U.S. adults delayed or avoided medical care because of concerns about COVID-19 (98). Most testing methods for COVID-19 focused on RT-qPCR, an effective method for detecting very small amounts of SARS-CoV-2 RNA. However, such molecular tests typically are performed by centralized high-complexity laboratories with special equipment and scaling to meet demands of public health testing has been a major challenge globally (99). During the COVID-19 pandemic, the testing capacity of many public health systems was overwhelmed and a global shortage of testing reagents and supplies occurred (100). These major bottlenecks in testing led to turnaround times exceeding 5 to 10 days in some regions of the U.S., making such tests unreliable in preventing disease transmission (99). Over-the-counter diagnostic tests, such as rapid antigen detection assays, are intrinsically quicker and less laborious than RT-qPCR. However, antigenic assays rely on individuals correctly performing self-tests and reporting positive results and are considered less accurate than RT-qPCR, especially for asymptomatic subjects (11, 12). These issues led to underreported cases, along with increases in false positive and false negative results (101).

Shortly after SARS-CoV-2 was reported to be present in human waste (102), monitoring the virus in wastewater gained attention as an effective epidemiological tool for tracking spatial and temporal dynamics of COVID-19 at the community level (22, 29–35). A few studies employing WS detected community transmission of SARS-CoV-2 prior to onset of new COVID-19 cases (31, 103, 104), suggesting longitudinal wastewater analysis could be used to detect viral shedding of infected (symptomatic and asymptomatic) individuals and identify trends sooner than clinical case reporting. However, it has been suggested that WS serves as a true early warning system only when community incidence of COVID-19 is low and clinical testing in the region of interest is scarce or deficient (105). Furthermore, due to the nature of analyzing pooled samples, e.g., samples collected from a SCS, individual contributions to a positive sample cannot be determined, and WS is not as effectively employed for contact tracing as clinical testing of individuals. Hence, WS is best suited as a complement to clinical testing, serving as an independent indicator of disease prevalence (10).

High-throughput molecular methods for microbiome analysis, e.g., DNA metagenomics and RNA metatranscriptomics, and SNV analysis of pathogens in wastewater allows microbiome comparisons between regions and detection of sources of infection and transmission dynamics (36–38, 106, 40). However, complete microbiome analysis of wastewater for community surveillance is very much in its infancy. With continued emergence of new SARS-CoV-2 variants, it is increasingly evident that COVID-19 will not be eradicated globally. While human-to-human interaction is the major route of transmission, and wastewater testing can elucidate community epidemiology, understanding the possible role of climatic and weather processes in accelerating such interactions is also an important component. Elucidating the role of environmental parameters and how they interact with the microbiome can be very helpful in developing a climate-informed understanding, translatable to predictive models for estimating risk of the infection in humans.

### Detection and characterization of SARS-CoV-2.

**FIG 7:**
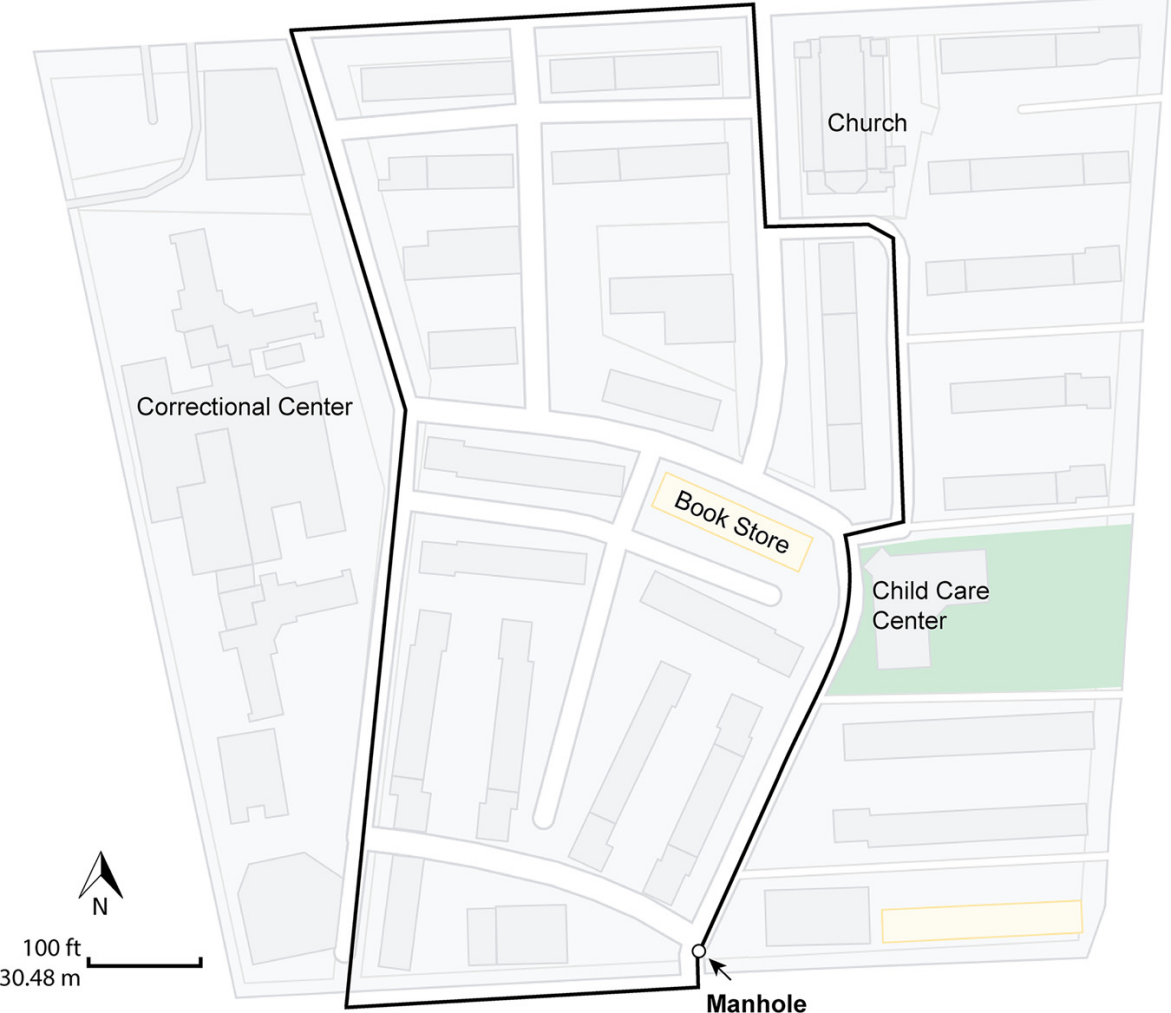
Map of sewage collection system. Map shows location of manhole where wastewater samples were collected. Black line indicates boundary of the area serviced by the sewer collection system. Arrow indicates location of manhole where samples were collected. The manhole is downstream of the area serviced, and wastewater flows from north to southeast. Scale bar corresponds to 100 ft (30.48 m).

In the study reported here, longitudinal wastewater analysis was employed for community surveillance (Fig. 1), with a manhole (Fig. 7) selected for sample collection because it offers distinct advantages over testing at a wastewater treatment plant (WWTP). SCSs are designed as tree networks, with the community (homes, schools, businesses, industries, etc.) flowing raw sewage and excess water into the network, which eventually feeds downstream into a local WWTP. Sampling at an individual manhole provides better understanding of how microbial communities are associated, distributed, and vary temporally within a geographically defined area, without being diluted with sewage from other geographic areas or other kinds of wastes, e.g., industrial. Generally, the amount of SARS-CoV-2 RNA detected in the community wastewater showed a pattern similar to the number of reported COVID-19 cases in the study area (Fig. 2). Previous studies have reported detection of an increased RNA load in wastewater prior to manifestation of positive cases, *ca.* 2 and 8 days (22), and prior to other pandemic indicators, e.g., COVID-19 hospital and intensive care unit administrations, up to 9 days (107). However, shedding of SARS-CoV-2 in feces is variable both in viral load and time course (108). Furthermore, the population served by this SCS represents *ca.* 3% of the total population covered by the epidemiological reports. It is also worth noting that the rate of clinical COVID-19 testing in MD varied in frequency throughout the study (81) and, despite the likelihood of increased number of reported COVID-19 cases reflecting active spread of the virus, the potential for a confounding influence of variations in testing frequency must be considered (109), which complicates calculating correlations between RNA concentration and number of infected individuals. Therefore, calculation of number of COVID-19 cases versus SARS-CoV-2 RNA concentrations was not done. However, RT-qPCR data are useful to assess trends in abundance of SARS-CoV-2 RNA detected in the SCS. Excluding the measurement above ROQ, the highest concentration of SARS-CoV-2 RNA detected over the course of the 48-week study (7.80 × 10^5^ N copies/L) was similar to other studies in the United States. employing RT-qPCR to detect SARS-CoV-2 RNA in raw wastewater influent, with *ca.* 10^5^ or 10^7^ N copies/L reported (22, 35).

Targeted amplicon sequencing for SARS-CoV-2 for variant calling among population-based samples, such as wastewater, compared with SNV analysis of single isolates recovered from patients and the environment, results in a consensus sequence that will identify the most prevalent genetic mutations within a sample. However, the resulting consensus has the potential to mask minor variants or merge mutations from multiple variants into a single sequence. Nonetheless, population-based variant calling in wastewater has recently been used to track viral evolution occurring in large WWTPs (110, 111). Yet, only a few studies have used it for analysis at the small community level, e.g., single building (25) or university dormitory (26). Those studies and others reported viral concentrations of *ca.* 10^6^ genome copies/L to be sufficient for sequencing SARS-CoV-2. Using methods reported here, we successfully employed targeted amplicon sequencing for detection and characterization of SARS-CoV-2 in wastewater samples with viral concentrations as low as 10^3^ N copies/L. However, calculations across studies are variable and depend on sample collection and preparation methods, as well as the sequencing method used, and, therefore, are not directly comparable. We were not able to detect SARS-CoV-2 using shotgun RNA metatranscriptomics, even when viral concentrations were relatively high (7.80 × 10^5^ N copies/L). That is, SARS-CoV-2 detection was successful when more sensitive methods were used, i.e., targeted amplicon sequencing and RT-qPCR. Spurbeck et al. (25) employed RT-qPCR, targeted sequencing, and untargeted metatranscriptomics to examine the local dynamics of SARS-CoV-2 strains and identify other pathogens circulating in the community, and concluded that alternative sequencing approaches may be required to consistently detect SARS-CoV-2 for biosurveillance, namely, due to low abundance of viral RNA compared to total RNA present in wastewater. Hence, multiple methods should be considered for wastewater surveillance.

On January 12, 2021, the first observation of the alpha variant (B.1.1.7) was reported in Maryland (112). Interestingly, this is around the time that a dramatic increase in the number of cases was observed in the study area. Targeted amplicon sequencing (Fig. 3) confirmed the presence of genetic mutations associated with this variant and others. An increase in SARS-CoV-2 RNA was detected in the wastewater samples in early February 2021, and SNVs commonly associated with the B.1.351 (Beta) variant were also detected. In April 2021, SNVs associated with B.1.1.7 (Alpha) were detected along with genetic mutations associated other SARS-CoV-2 lineages prior to the February increase and on March 23, 2021, indicating that multiple variants may have been circulating in the region. Similar observations have been made during genomic surveillance of SARS-CoV-2 in Delaware, USA (113), showing how quickly Alpha variants spread throughout a community. These data, coupled with what is known about increased transmissibility of B.1.617.2 (Delta) and B.1.1.529 (Omicron), highlight the importance of this type of proactive genomic surveillance.

Although most mutations may be deleterious or neutral, a few have the potential to affect viral function, potentially altering infectivity and/or disease severity (76). The SARS-CoV-2 spike protein recognizes host cells and is the main target of the human immune response, and some of the protein mutations have been linked to heightened infectivity and the ability to evade infection-blocking antibodies (114). During the onset of COVID-19, two major locally transmitted outbreaks occurred in China, one in Wuhan from December 2019 to April 2020, another in Beijing-Xinfadi in June 2020 (115). Interestingly, SARS-CoV-2 sequences recovered from Beijing-Xinfadi can be distinguished by a single nucleotide at position 241, a conserved pyrimidine nucleotide located in the loop region of stem-loop 5B of the 5′-untranslated region (116), compared with those collected in Wuhan. Viral isolates from patients and the environment generally encode uridine, whereas the viruses isolated in Wuhan encode a cytidine nucleotide at the corresponding position (115). In this study, the C241T variation was observed at all time points. The S protein mutation D614G, detected in samples, collected on February 11 and 17, 2021 and March 23, 2021, is an important genetic mutation associated with increased SARS-CoV-2 infectivity (117). Interestingly, detection of S:D614G coincided with the detection of higher SARS-CoV-2 RNA concentrations (Data set S2). The P681H mutation, detected at four time points in this study—February 11, 2021, March 10 and 23, 2021, and April 15, 2021—is common to B.1.1.7 isolates and has been characterized as part of the spike S1/S2 cleavage site, with potential to enhance viral cell entry (118). Other spike SNVs detected in this study, namely, T716I, S982A, and D1118H, have also been associated with natural mutations observed in B.1.1.7 isolates that show greater transmissibility than wild-type SARS-CoV-2 isolates (119). However, it is worth noting that the spike mutation K417N was detected in the sample collected on February 17, 2021, which corresponds to the time point with highest concentration of SARS-CoV-2 RNA detected via RT-qPCR (Fig. 2), is commonly associated with B.1.351 isolates of high concern because they have potential to compromise neutralization generated by previous infection or vaccination (120, 121).

### Environmental COVID-19 risk score analysis and wastewater surveillance.

Usmani et al. (6) reported transmission of SARS-CoV-2 in the human population increased when the ambient air temperature (AT) is out of the comfort zone (17°C to 24°C). When temperatures are uncomfortable, i.e., outside the comfort zone, human behavior in ambient and built environments is related to increased COVID-19 transmission (Fig. S1). In addition to AT, increased transmission of SARS-CoV-2 was reported in cold and dry environments. In this study, the association of AT and DPT with incidence of COVID-19 in the human population was related to detection of SARS-CoV-2 in wastewater (Fig. 2). Results showed a statistically significant (*P* < 0.1) positive correlation between environmental risk scores and reported cases. When the environmental risk was greater than 0.5, more COVID-19 cases were reported, and SARS-CoV-2 was detected 88% of the time (via RT-qPCR) in wastewater. *Per contra*, when the risk score dropped below 0.5, only a few COVID-19 cases were reported, and SARS-CoV-2 RNA was rarely (31% of the time) detected in wastewater and was at low abundance. Hence, incidence of SARS-CoV-2 in the human population examined here appears associated with AT and DPT, and hence, WS as a COVID-19 risk score can be a useful public health tool. Because the environmental predictive risk model has a spatial resolution of 4 km × 4 km, the proposed model is best suited to identify regions with heightened environmental risk. In turn, WS can be used to provide ground truth for presence of SARS-CoV-2, along with other pathogens, and determine potential for disease transmission at a local level, e.g., community or neighborhood. Employing such an approach can reduce human labor and identify targeted regions where there may be a need for WS to capture finer spatial resolution of disease transmission, thereby serving as an effective tool to mitigate disease.

### Wastewater microbiome.

#### (i) Microbial diversity.

DNA metagenomics can be used to detect and identify the bacteria, virus, fungi, and protists comprising the microbial community of environmental samples (39), with viability or infectious potential of the detected microorganisms requiring additional analyses of metabolic activity. RNA metatranscriptomics is useful for detecting RNA viruses, but can also give insight into the functional profile of the microbiome (122). Both DNA metagenomics and RNA metatranscriptomics have been used to explore the microbiome of wastewater (36–38, 123).

Bacteria dominated the microbiome of wastewater samples in this study (Fig. S4), consistent with prior observations of microbiomes of samples collected at WWTPs (36, 38, 124). Similarly, the bacterial phyla (Fig. 4A) were those commonly detected in WWTPs (124), namely, *Firmicutes*, *Proteobacteria*, and *Bacteroidetes*, as the most abundant. Similarly, *Ruminococcaceae*, a microbial family detected in most WWTP samples (124), was present in all samples. However, *Bacteroidaceae*, *Bifidobacteriaceae*, *Comamonadaceae*, and *Lachnospiraceae*, were also dominant in the wastewater microbiome. Genera (Fig. 4B) included *Bacteroides*, *Bifidobacterium*, *Acidovorax*, Acinetobacter, Pseudomonas, *Alistipes*, and *Prevotella*, similar to reports of other investigators (36, 38, 124). Previous studies suggest *Trichococcus* spp. are dominant members of the microbial community in urban sewers (125), but species of this genus were present at lower RA in this study. During a metagenomic survey of wastewater in the United Kingdom, *Arcobacter* and *Aeromonas* were identified as predominant wastewater indicators (126) and those genera were detected in this study.

Several studies investigating the wastewater microbiome have been published. However, most of those studies use partial 16S rRNA gene sequencing, which usually does not allow microbial profiling to lowest taxonomic resolution, i.e., subspecies (39). Few studies detailed microbiome profiles of wastewater to species. Here, we employed SMS to profile the microbial composition of wastewater to subspecies (Fig. 4C). In addition, archaea, protists, fungi, and viruses were detected and identified (Fig. 4D). Protozoan species common to wastewater, such as *Pseudoperonospora cubensis* and *Paramecium biaurelia*, are environmental protists associated with infection in plants (127) and frequent symbionts of green algae in the aquatic environment (128), respectively. Fungi are ubiquitous in the environment, coexisting and interacting with other microorganisms and regulating a range of ecological functions. Fungi also are an important group of microorganisms found in wastewater, aiding organic decomposition (129). However, some fungal genera commonly detected in WWTPs are opportunistic pathogens for humans and plants (130), including *Olipidium*, Aspergillus, *Candida*, and *Penicillium*, and these were detected in this study. Interestingly, *Epichloe sylvatica*, found previously to be associated with biofilm in WWTPs (36), was the most frequent fungal species detected in all samples examined in this study.

#### (ii) Virome.

Traditionally, microbial communities have been defined using culture dependent methods to detect and enumerate microorganisms. However, it is estimated that the vast majority of prokaryotic genospecies remain uncultured. Accordingly, metagenomics and metatranscriptomics obviate the need to isolate and culture microorganisms. Furthermore, molecular methods are used to profile viral communities. In the current study, a combination of molecular methods, i.e., RT-qPCR, targeted amplicon sequencing, DNA metagenomics, and RNA metatranscriptomics, were used to detect and identify the wastewater virome. DNA viruses were detected in all samples, with *Papillomaviridae* and *Polyomaviridae*, human viruses, detected in samples positive for SARS-CoV-2 (Fig. 4D). The most frequently detected RNA viruses included tomato brown rugose fruit virus, pepper mild mottle virus, cucumber green mottle mosaic virus, tomato mosaic virus, tobacco mild green mosaic virus, tropical soda apple mosaic virus, tomato mottle mosaic virus, and melon necrotic spot virus (Fig. 5A). This viral composition was reported in a study of a WWTP in southern California during the COVID-19 pandemic (123). Results reported here show RNA metatranscriptomics is effective in detecting the dominant viruses in circulation, in agreement with prior research (25).

#### (iii) Resistome and virulome.

Metagenomic sequencing has been used to explore AMR trends in anthropogenically impacted environments (41) and to detect wastewater associated enteric pathogens (36, 95). However, viability or infectious potential of detected microorganisms requires additional analysis of metabolic activity. In addition to detecting RNA viruses (25, 43), high-throughput sequencing of cDNA for RNA analysis, i.e., RNA sequencing, provides insight into gene expression (44) and has been used for metatranscriptomic analysis of wastewater (45). In the current study, untargeted RNA metatranscriptomic sequencing was employed successfully to analyze the active microbial community structure of wastewater samples, including functional activities, active pathways, and AMR and VF associated gene expression (Fig. 5). RNA viral genes are replicated, expressed, and assembled in association with living host cells (46). It has been suggested that SARS-CoV-2 can remain viable in sewage up to 4.3 days (47) and other coronaviruses may remain viable in aqueous matrices for over 1 year (48). In the current study, evidence of viral RNA genome replication was detected in 50% of the wastewater samples examined, suggesting infectious potential of RNA viruses in wastewater.

AMR is a growing global threat, claiming 700,000 deaths per year, further complicated by the COVID-19 pandemic, (49). Antibiotics have been remarkedly successful in treating cobacterial infections associated with COVID-19, but these drugs do not work on viruses, including coronaviruses or influenza (50). In this study, the greatest number of AMR associated genes, namely those of the Macrolides, were detected in the sample collected on February 17, 2021, which was the week prior to the observed increase in SARS-CoV-2 RNA in wastewater. Interestingly, no AMR associated genes were detected in the sample collected the week following the increase in detected SARS-CoV-2 RNA, February 17, 2021. A similar pattern was observed with genes coding for VF, where an increase in VF was observed the week prior to the observed increase in SARS-CoV-2 RNA. VF associated with *Bacteroides* was dominant in these samples. *Bacteroides* are important members of the gut microbiome and have been linked to dysbiosis and the fecal-viral load during COVID-19 infection (51). While no attempts were made to associate clinical data to the resistome or virulome of wastewater detected and identified in this study, the results support previous observations that antibiotic use may be associated with progression of COVID-19 and bacterial coinfection (52), and may shed light on changes to the human fecal microbiome during COVID-19 infection (53), with respect to abundance of opportunistic pathogens and the VF carried.

### Quantitative comparative metagenomics.

Detection of wastewater associated pathogens by employing molecular techniques has been done at WWTPs (36, 38, 124) and used for source tracking of fecal pollution (126, 95, 54). However, a major hurdle for WS is communication of the results and their interpretation to public health officials. Metagenomic surveys are typically presented as the RA of sequencing reads, not a meaningful parameter for microbiota quantification and can be biased, depending on the experimental protocol employed (55). To circumvent these issues, we included an *in situ* control to the DNA metagenomics, allowing absolute cell number quantification, a recent technique increasingly cited in literature (56, 58). Presenting metagenomic sequencing results as cell numbers provides more precise microbial load estimates and permits normalization of studies. To present the utility of this application, the cell number of microorganisms detected in wastewater samples, e.g., pathogens and bacteria commonly employed as indicators of human fecal contamination (95, 59), is presented in Table 1, and can be used as a comparative baseline for metagenomic investigations employing this technique.

### Wastewater microbiome and SARS-CoV-2 surveillance.

While COVID-19 is generally considered to be primarily a respiratory illness, gastrointestinal symptoms, e.g., diarrhea, vomiting, nausea, or abdominal pain, are commonly associated with this disease (60). Therefore, gut microbiota have been linked to a variety of COVID-19 risk factors for infection and the gut microbiome is a potential therapeutic target for many other diseases (61). Emerging evidence suggests SARS-CoV-2 can infect the gastrointestinal tract directly (63).

Using DNA metagenomics, we aimed to evaluate if changes in the wastewater microbiota could be associated with prevalence of SARS-CoV-2 RNA in a specific community. The inclusion of an *in situ* control allowed correlations to be made between quantified microbial load (cells/L) and concentration of SARS-CoV-2 RNA (cells/L) detected in wastewater samples (Fig. 6). A similar study used Nanopore 16S rRNA sequencing to profile the microbiome of wastewater samples during a COVID-19 outbreak in Chile and showed correlation between RA, namely percentage, of both bacteria and SARS-CoV-2 (64). Strikingly, members of families *Lachnospiraceae* and *Rumnococcaceae* and genus *Alistipes* were detected at increased abundance following a COVID-19 outbreak, an observation supported by findings presented here. Gallardo-Escárate et al. (64) reported strong association with *Prevotella*, *Bacteroides*, *Aeromonas*, *Sulfurospirillum*, *Arcobacter*, *Tolumonas*, *Citrobacter*, *Zoologea*, and *Janthinobacterium*. In the current study, a similar correlation was observed for members of these taxa, but below our threshold of confidence. Statistical differences for these taxa may result from the sequencing method used, i.e., 16S rather than SMS (39). Because the wastewater microbiome is dominated by human gut bacteria, similar investigations done in different geographical regions, including areas where the diet differs, e.g., western versus eastern diets, which can influence the gut microbiome, are required to confirm these observations.

Here, bacterial genera with co-occurrence correlated with SARS-CoV-2 include *Bifidobacterium*, *Leclercia*, *Pyramidobacter*, *Tannerella*, *Massilimaliae*, and *Erythrobacter* (Fig. 6A). Most of the species determined to have significant co-occurrence correlation with SARS-CoV-2 were bacteria commonly found in the human gut (Fig. 6B). These include Paraprevotella xylaniphila, Phascolarctobacterium succinatutens, and Slackia piriformis, and also various *Bifidobacterium* spp. (*B. adolescents*, B. catenulatum, B. pseudocatenulatum, and B. pseudolongum), *Bacteroides* spp. (*B. caecimuris*, *B. nordii*, and *B. rodentium*), and *Prevotella* spp. (*P. lascolaii*, P*. marseillensis* and *P. pectinovora*), supporting the hypothesis that the resident microbial communities of the gastrointestinal and respiratory tracts can act as modulators of local and systemic inflammatory activity, e.g., the gut-lung axis (65). Remarkably, several opportunistic species shown to have significant correlation with SARS-CoV-2 RNA in wastewater, including Bordetella bronchiseptica (66), Enterobacter cloacae (67), Leclercia adecarboxylata (68), and Pseudomonas monteilii (69). These have been identified clinically as coinfecting microorganisms during COVID-19. Co-occurrence of archaea, protozoa, fungi, and DNA viruses was analyzed (Fig. 6C), but results of combined kingdom analysis were inconclusive. Additional investigation is required to elucidate these complex interactions.

### Challenges and future directions.

Observations in this study were characterized using samples limited in number, yet illustrative of an outbreak in a single location and representing temporal shifts in wastewater microbial communities during an increase in COVID-19 cases. The data presented in this study were not normalized to flow rate or fecal and urine load, which have shown to be important in comparing microbial loads in wastewater (70–72). Additional studies are needed to characterize fully the core wastewater microbiome and establish a comparative baseline to verify microbial shifts between outbreak and nonoutbreak conditions, and establish public health significance. The wastewater microbiome of samples collected from a manhole may differ from wastewater samples collected from other SCSs, e.g., hospitals, prisons, nursing homes, WWTPs, etc. In additional to more samples and other locations, different seasons of the year need to be studied. In particular, such investigations will shed light on significant shifts in SMS and qRT-PCR results relative to COVID-19 outbreak size and duration, as well as incidence of other communicable diseases, notably influenza.

With respect to methods used in this study, because of the complexities of detecting SARS-CoV-2 in the environment, the decision was to centrifuge samples after collection to remove large debris and then employ ultrafiltration. It is possible that centrifugation removed larger microorganisms and those associated with sediment thereby influencing the microbiome profile. Hence, comparison of sample processing, sequencing, and bioinformatics is in progress.

Implementation of SMS for WS requires access to laboratories, trained personnel, bioinformatics support, and infrastructure for data storage. SMS can take longer to perform and currently is more expensive than traditional microbiological testing. The information provided, however, will prove valuable for public health.

Many short-read sequencing technologies rely on clonal PCR for signal detection. However, new technologies with long reads and single-molecular sequencing, such as those of Pacific Biosciences and Oxford Nanopore, are proving useful (42). Recently it has been shown that rapid variant identification of SARS-CoV-2 can be achieved in near real-time using a miniature-sized and field-deployable Oxford Nanopore MinION sequencing device (73) and also for microbiome analysis (74). Similarly, single cell sequencing has emerged as a useful molecular tool to identify genetically diverse viral genomes within single infectious units (75); its application in WS would help navigate variant calling in samples of a pooled population.

It is important to note that municipalities across the world now track SARS-CoV-2 in wastewater, and the addition of SMS will provide detailed characterization of wastewater microbiome and detection of infectious agents circulating within local populations. This is a crucial step in preparing for future epidemics and pandemics. This approach can also be used to monitor an array of public health indicators, e.g., obesity (77), consumption of alcohol, illicit drugs and tobacco, exposure to hazardous chemicals and pharmaceuticals (23, 24), and detection of sexually transmitted disease etiological agents (78, 79). In the meantime, SMS can be usefully applied to clinical and WS practices directed at both community and global public health.

### Conclusion.

Wastewater surveillance (WS) is accomplished by WWTPs in many municipalities globally to track SARS-CoV-2. In the study reported here, detection was achieved using RT-qPCR but was also combined with advanced molecular sequencing to characterize SARS-CoV-2 genetic mutations (targeted amplicon sequencing) and profile the complete microbiome (DNA metagenomics and RNA metatranscriptomics) of wastewater samples collected from a sewage collection system (a manhole). The microbial communities of the wastewater were associated, distributed, and varied temporally within a defined geographical area. Trends observed in this study are for a single location, but when environmental COVID-19 risk prediction was combined with the WS results, it was observed that after introduction of SARS-CoV-2 into a community, climatic conditions, namely, temperature, is associated with viral transmission. WS serves as a useful indicator of disease prevalence, but results show early detection, characterization of SARS-CoV-2, and co-infections in a community can be determined using microbiome profiling of wastewater. Ongoing research will integrate clinical case reports of gastrointestinal and other communicable diseases prevalent in a community, in addition to COVID-19, to assess whether changes in the wastewater microbiome are indicative of community health and if reliable biomarkers can be detected that are applicable to public health.

## MATERIALS AND METHODS

### Site description.

WS was carried out by monitoring a component of a SCS in Maryland, USA (Fig. 7). The exact location of the study has been masked to protect the privacy of persons living in the area. Total daily number of reported COVID-19 cases among Maryland residents within the ZIP code where the study was conducted, which has an estimated population of *ca*. 22,000 (80), was retrieved from the Maryland Department of Health (81). The cumulative number of positive COVID-19 cases is presented in the supporting information (Data set S1). At the beginning of the study period, the SCS serviced 45 buildings with 669 housing units, equating to a population density of roughly 760 residents. The average daily wastewater flow (*ca.* 17,610 L per day) was estimated from the average daily accumulative water flow of all buildings serviced by the SCS between October 26 and November 25, 2021, using a conversion factor of 80%. Justification of the flow projection based on water consumption in the serviced community is supported by calculations detailed elsewhere (82).

10.1128/mbio.00591-22.7 DATA SET S1 Cumulative number of positive COVID-19 cases among Maryland residents within the ZIP code where the study was conducted. Download Data Set S1, XLSX file, 0.02 MB. Copyright © 2022 Brumfield et al. 2022 Brumfield et al. https://creativecommons.org/licenses/by/4.0/ This content is distributed under the terms of the Creative Commons Attribution 4.0 International license.

### Sample collection.

Composite samples were collected weekly from a manhole downstream of the SCS between December 20, 2020 and November 16, 2021 (*n* = 48). During each sampling event, an automated composite sampling unit (Teledyne ISCO, Lincoln, NE, USA) was prepared in the morning with ice surrounding the collection jar. Samples (60 mL) were collected at 15-min intervals for 24 h, totaling 96 individual sampling attempts and a composite volume of 5.76 L. Temperature of the composite samples was recorded at time of retrieval and is provided in the supporting information (Data set S2). Samples were homogenized manually, and an aliquot (110 mL) was pasteurized in a water bath (Polyscience, Niles, IL, USA) at 60°C for 30 min, transported to the laboratory on ice, and processed the same day. To remove larger debris, 45 mL were transferred to a sterile polypropylene 50 mL conical Falcon tube (Corning, Glendale, AZ, USA) and centrifuged at 7,500 RCF for 10 min at 4°C. The resulting supernatant was transferred to a clean conical tube and concentrated using InnovaPrep Concentrating Pipette Select with Ultrafilitration PS Hollow Fiber pipette tips (nominal molecular weight limit of *ca.*100 to 120 kDa; InnovaPrep, Drexel, MO, USA), following the manufacturer’s recommendations for “Wastewater Application Note, Revision B” (83). InnovaPrep Wet Foam Elution was stored in DNA/RNA shield (Zymo Research, Irvine, CA, USA), following the manufacturer’s specifications, at −80°C until nucleic acid was prepared (<48 h). Detection of SARS-CoV-2 RNA was done on all the samples that were collected and six samples were selected for microbiome analysis.

### COVID-19 risk assessment.

Usmani et al. (6) hypothesized transmission of SARS-CoV-2 occurs in the human population when the ambient AT rises above or falls below the “comfort zone” (17°C to 24°C) and transmission of the virus thereby increases (Fig. S1). This phenomenon is the result of changes in human behavior in the ambient and built environment, namely, when temperatures become uncomfortable. That is, when AT is within the comfort zone, there will be a decrease in COVID-19 transmission. Furthermore, an increase in aerosolization of SARS-CoV-2 occurs in cold and dry environments. For example, an increased number of COVID-19 cases were reported in the northern part of the U.S. during winter months, compared with other seasons. In the current study, we sought to evaluate the feasibility of employing this hypothesis as a predictive COVID-19 risk score model and demonstrate its potential use with WS.

To examine this model, details of which are described elsewhere (6), environmental COVID-19 risk scores were predicted along with epidemiological and experimental data obtained throughout the study. The difference between AT and dew point temperature (DPT) was used as an indication of moisture availability in the atmosphere, an observation described previously to impact viral survival as a seasonal factor in influenza and poliomyelitis (84, 85). Briefly, DPT, defined as the temperature at which water vapor condenses to form liquid droplets large enough to settle quickly on Earth’s surface due to gravity, is directly proportional to moisture content in the air. That is, low DPT indicates lower moisture in the air and high DPT suggests high moisture content. Hence, DPT must be less than or equal to AT. The difference between AT and DPT indicates moisture saturation of the ambient air, and the difference is inversely proportional to air moisture saturation. If the difference between AT and DPT is zero, then the air is considered fully saturated. As the difference between AT and DPT increases, the air becomes less saturated. In summary, DPT indicates specific moisture in the air, while the difference between AT and DPT can be used to determine moisture saturation in the ambient air.

Daily AT and DPT were obtained from the Oregon State University Parameter-elevation Regressions on Independent Slopes Model (PRISM) climate group products at a resolution of 4 km × 4 km (86). Prediction of environmental COVID-19 risk was computed weekly using AT and DPT as model input and quantified according to the hypothesis, where deviation of AT outside the comfort zone was calculated for the previous 2 weeks. The number of days with a negative DPT and when the difference between AT and DPT was greater than 5°C was also determined for the same time interval. Variables were determined using a scale of 0 to 4, with 4 being the maximum value. Environmental risk of COVID-19 was determined from the normalized sum of quantified variables with equal weight. That is, the risk model output varies between 0 and 1, with 1 being the highest risk of transmission. Following environmental risk assessment, weekly risk score values were compared with RT-qPCR observations of SARS-CoV-2 in wastewater samples.

### Quantitative reverse transcriptase PCR for detection of SARS-CoV-2 RNA.

#### (i) RNA purification.

To serve as internal control (IC) for the RT-qPCR assay, 25 μL of MS2 Bacteriophage, target titer of 1.0 × 10^3^ PFU/mL, (ZeptoMetrix, Buffalo, NY, USA), was added to 115 μL InnovaPrep Wet Foam Elution. Total RNA was prepared from wastewater concentrates containing MS2 Bacteriophage, employing the QIAamp Viral RNA minikit (Qiagen, Germantown, MD, USA), following the manufacturer’s instructions for use on the automated QIAcube Connect platform. RNA extracts were stored in LoBind microcentrifuge tubes (Eppendorf, Hamburg, Gernamny) at −80°C prior to RT-qPCR amplification (<48 h).

#### (ii) Reference RNA materials.

The SARS-CoV-2 Research Grade Test Material (RGTM 10169; National Institute of Standards and Technology, Gaithersburg, MD, USA), consisting of two synthetic RNA fragments from the SARS-CoV-2 genome (including SARS-CoV-2 sequences 25949 to 29698 and 12409 to 15962 of isolate USA-WA1/2020) in a background of 5 ng/μL human Jurkat RNA, was diluted over six serial log dilutions with nuclease-free water for use as calibration standards. RNA template of SARS-CoV-2 Nucleocapsid Phosphoprotein (N protein) encapsulated in the MS2 bacteriophage construct (PerkinElmer, Waltham, MA, USA) was prepared following the manufacturer’s instructions for use as positive control (PC). Single-use aliquots of reference RNA were stored in LoBind microcentrifuge tubes (Eppendorf, Hamburg, Gernamny) at −80°C.

#### (iii) RT-qPCR amplification.

The New Coronavirus Nucleic Acid Detection Kit v.7.0 (PerkinElmer, Waltham, MA, USA) was employed using the QuantStudio 3 System (Thermo Fisher, Waltham, MA, USA), for multiplexed detection of SARS-CoV-2 N protein and IC, following the manufacturer’s specifications. The New Coronavirus Nucleic Acid Detection Kit also allows for detection of SARS-CoV-2 Open Reading Frame 1ab (ORF1ab). However, under the methods detailed below, controlled spike-in experiments have suggested that N protein provides a more accurate estimation of SARS-CoV-2 abundance in wastewater. Internal lab results, including multiple temporal samples from different locations, have shown that wastewater samples presenting detection of ORF1ab alone (ORF1ab positive and N negative) were extremely rare. Hence, N protein was used as the representative SARS-CoV-2 marker for subsequent analysis. Amplification efficiency (*AE*) for each reference DNA calibration model was calculated as follows: *AE* = 10^-1/*slope*^ - 1. The lower limit of quantification (LLOQ) was determined by signal-to-noise ratio of 10:1 and approximated using residual standard deviation (SD) of regression, defined by: $\mathrm{LLOQ}=10\frac{\mathrm{SD}}{-\mathrm{slope}}$. The upper ROQ was defined as the highest calibration standard included in the standard curve (5 × 10^6^ N copies per reaction). Calibration model performance of each RT-qPCR run is provided in the supporting information (Data set S2). Following each run, log florescence thresholds were set manually, and melt curve analysis was done to identify spurious amplicons that could confound data interpretation (no spurious amplicons detected). Quantification cycle (Cq) values were exported to Excel (Microsoft, Redmond, WA, USA) for further analysis.

#### (iv) Quality control.

Method extraction controls (positive, PC; negative, NC; and internal, IC) were prepared using the New Coronavirus Nucleic Acid Detection Kit (PerkinElmer, Waltham, MA, USA) to monitor sample processing. Synthetic SARS-CoV-2 N protein RNA template encapsulated in MS2 bacteriophage (PerkinElmer, Waltham, MA, USA) was included as PC. For IC, bacteriophage MS2 was used to monitor the process from nucleic acid extraction to fluorescence detection for amplification inhibition. Failure to detect PC resulted in an invalid run for all samples. Samples negative for IC, suggestive of amplification inhibition, or positive for NC, suggestive of potential contamination, were discarded from further analysis.

### Complete microbiome analysis employing next generation sequencing.

A total of six samples were selected for complete microbiome analysis, including shotgun DNA metagenomic and RNA metatranscriptomic sequencing, and targeted amplicon sequencing for identification and characterization of full SARS-CoV-2 genome.

#### (i) Nucleic acid preparation.

Total RNA was prepared from InnovaPrep Wet Foam Elution using QIAamp Viral RNA minikit, as mentioned previously. To serve as an *in situ* control for DNA sequencing, the ZymoBIOMICS High Microbial Load Spike-in Control I (Zymo Research, Irvine, CA, USA) was added to each sample, per manufacturer’s specifications. Total DNA was prepared from 250 μL of InnovaPrep Wet Foam Elution using the DNeasy PowerSoil Pro Kit (Qiagen, Germantown, MD, USA), following the manufacturer’s instructions for use on the automated QIAcube Connect platform. Nucleic acid concentrations were determined using Qubit Broad Range Assay Kits (Thermo Fisher Scientific, Waltham, MA, USA) on an Invitrogen Qubit 4.0 Fluorometer (Thermo Fisher Scientific, Waltham, MA, USA) for RNA and double-stranded DNA, respectively.

#### (ii) Targeted sequencing for identification of full SARS-CoV-2 genome.

Following detection and quantification of SARS-CoV-2 by RT-qPCR, excess cDNA of selected samples and sample processing controls (method extraction blank and synthetic SARS-CoV-2 RNA) was used as input to establish mutation profiles. Sequencing libraries were prepared using the Swift Normalase Amplicon SARS-CoV-2 Panel kit (Swift Biosciences, Ann Arbor, MI, USA), following the manufacturer’s instructions. The Swift Normalase Amplicon SARS-CoV-2 Panel involves multiplex PCR technology that utilizes multiple overlapping amplicons. Using tiled primer pairs targeting the entire SARS-CoV-2 genome enables library construction from cDNA, even from low input quantities. The resulting libraries were quantified using Qubit dsDNA High Sensitivity assay kit (Thermo Fisher Scientific, Waltham, MA, USA) on an Invitrogen Qubit 4.0 Fluorometer (Thermo Fisher Scientific, Waltham, MA, USA), and sequenced 2 × 150 bp on an Illumina HiSeq4000 Instrument (Illumina Inc., San Diego, CA, USA).

General sequencing statistics for all samples and mean sequence quality distribution were measured by FastQC v.0.11.6 (87). Base-calling error probabilities (P) were evaluated using Phred Quality Score (Q), defined by: *Q* = -10*log*_10_(*P*). Using a previously defined read quality threshold (88), adapter and quality trimming of raw sequencing reads were done using Trimmomatic v0.38 (89) to ensure a Phred Quality Score of at least 17 for at least 80% of the read lengths, i.e., probability of correct base call was at least 98%. Primers used during panel amplification were trimmed using Primerclip v.0.3.8 (Swift Biosciences, Ann Arbor, MI, USA). Processed sequencing reads were aligned to the Wuhan-Hu-1 genome (NCBI accession no. NC_045512.2) using BBmap v38.70 (90), and variants were called using bcftools v.1.14 (91).

#### (iii) DNA metagenomics and RNA metatranscriptomics for complete microbiome analysis.

rRNA (rRNA) of abundant species (human, mouse, and rat) was reduced using Ribo-Zero Plus rRNA Depletion Kit (Illumina Inc., San Diego, CA, USA). Second strand synthesis of cDNA and preparation of RNA libraries was achieved using NEBNext Ultra II Nondirectional RNA Library Prep Kit (New England BioLabs, Ipswich, MA, USA), following the manufacturer’s instructions for use with Illumina chemistry. DNA libraries were prepared using Nextera XT DNA Library Prep Kit (Illumina Inc., San Diego, CA, USA). All sequencing libraries were quantified and sequenced using Illumina chemistry, as mentioned previously. A no template control (NTC), consisting of nuclease free water, and sequencing standard, i.e., ZymoBIOMICS^TM^ Microbial Community Standard (Zymo Research, Irvine, CA, USA), were included for quality control.

#### (iv) Microbiome taxonomic profiling.

For shotgun DNA metagenomics and RNA metatranscriptomics, adapter and quality trimming of raw sequencing reads was done as described previously. Unassembled metagenomic and metatranscriptomic sequencing reads were analyzed, as reported elsewhere (62, 92–95), using CosmosID-HUB Microbiome Platform (CosmosID Inc., Germantown, MD, USA) to achieve multikingdom microbiome analysis. Virulence and AMR associated gene profiling was done using RNA metatranscriptomic data through the CosmosID-HUB. Qualitative gene expression of detected Virulence and AMR associated genes is presented as the RA compared to ribosome binding molecular function (GO:0043022), which was detected at roughly equal abundance across all samples when normalized to copies per million reads. Additional information on the bioinformatics pipeline employed for taxonomic classification of sequencing reads can be found in the supporting information (Text S1).

10.1128/mbio.00591-22.1 TEXT S1 Taxonomic classification of metagenomic and metatranscriptomic sequencing reads. Download Text S1, DOCX file, 0.03 MB. Copyright © 2022 Brumfield et al. 2022 Brumfield et al. https://creativecommons.org/licenses/by/4.0/ This content is distributed under the terms of the Creative Commons Attribution 4.0 International license.

#### (v) Statistical analysis.

Microbiota detected in the NTC (*Pseudoxanthomonas* spp.) were subtracted from taxonomic profiling of DNA metagenomics; no microbiota were detected in the RNA metatranscriptomic NTC. For DNA metagenomics, microbial cell number of each taxon was normalized to the cell number of Imtechella halotolerans (Gram-negative) and Allobacillus halotolerans (Gram-positive), following the manufacturer’s specifications of the ZymoBIOMICS High Microbial Load Spike-in Control I (Zymo Research, Irvine, CA, USA). Quantification of the organism RA was defined as the proportion of unique organism-specific k-mers annotated by each database relative to the total number of unique sequencing reads generated for that sample. Analysis of community virulome and resistome were achieved by identifying virulence and AMR associated genes based on percent coverage as a function of gene-specific k-mer frequency in each sample.

The alpha diversity of bacterial communities associated with wastewater was compared among samples by calculating the Shannon and Simpson 1-D diversity indices and the Chao-1 richness index using Vegan v.2.5.7 (96). RA of bacterial taxa in each sample was used for principal coordinate analysis (PCoA) employing Bray-Curtis dissimilarity index, using CosmosID-HUB Microbiome Platform v.2.0 (CosmosID Inc., Germantown, MD, USA). Pair-wise Spearman-rank co-occurrence between concentrations of SARS-CoV-2 RNA (N copies/L) and microbiota (cells/L) was determined using phylosmith v.1.0.6 (57), which is based on methods described elsewhere (97). Upper and lower cutoffs for which rho values are not likely to have occurred by random chance were determined by calculating the pairwise Spearman rank co-occurrence for each variable with 1,000 permutation iterations. Significance for co-occurrence of each taxa and SARS-CoV-2 was determined using rho value cutoffs and *P* value (≤0.02).

### Data availability.

Sequencing data generated for all samples included in this study are deposited in the NCBI Sequence Read Archive database under BioProject ID PRJNA812772. Accession numbers for individual sample sequencing read libraries (SAMN2649443 to SAMN2649459) are provided in the supplementary information (Data set S4).
