# Supplementary material for: Microbiome Analysis for Wastewater Surveillance during COVID-19
Source: mBio. 2022 Jun 21;13(4):e00591-22. doi: 10.1128/mbio.00591-22 (PMC9426581; doi:10.1128/mbio.00591-22)
Supplement: FIG S1 [file mbio.00591-22-s0002.docx]

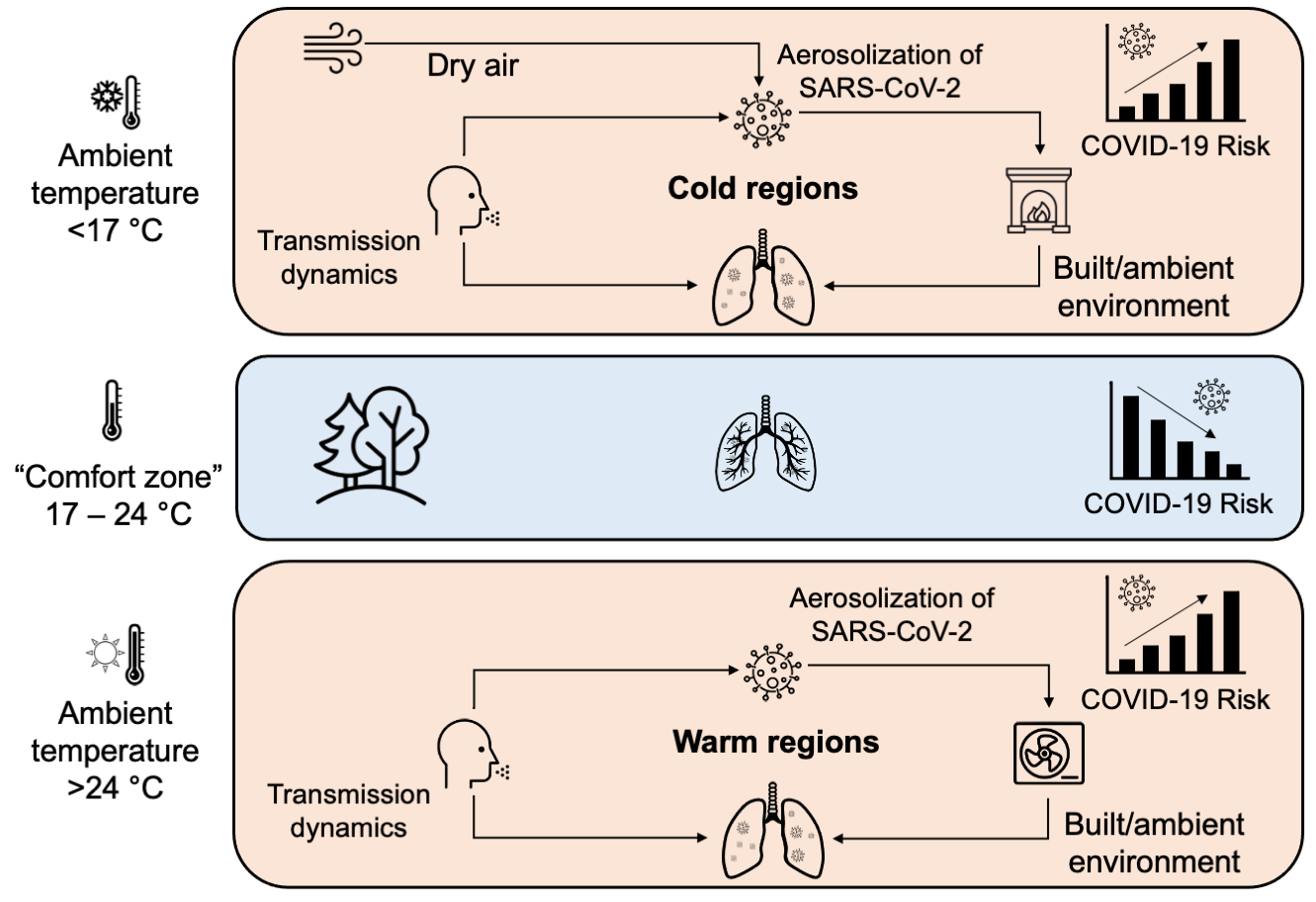


**Figure S1: Hypothesis for environmental COVID-19 risk prediction, adapted from Usmani et al. (Am J Trop Med Hyg 106:1-9, 2020,** [**https://doi.org/10.4269/ajtmh.21-0328**](https://doi.org/10.4269/ajtmh.21-0328)**).**
