# Supplementary material for: Microbiome Analysis for Wastewater Surveillance during COVID-19
Source: mBio. 2022 Jun 21;13(4):e00591-22. doi: 10.1128/mbio.00591-22 (PMC9426581; doi:10.1128/mbio.00591-22)
Supplement: FIG S2 [file mbio.00591-22-s0003.docx]

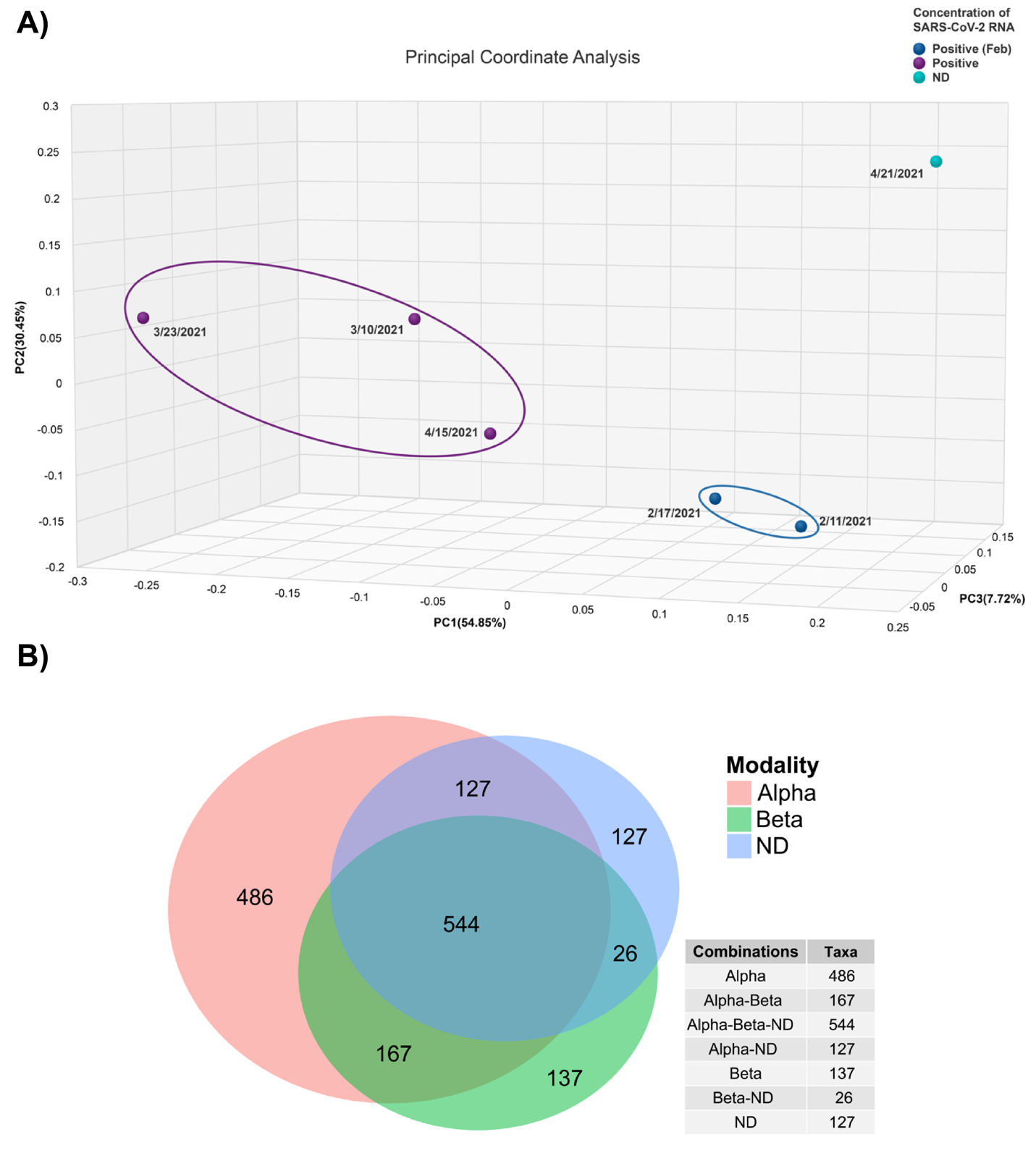


**Figure S2: Diversity of bacterial communities profiled in wastewater with respect to SARS-CoV-2.**

**Diversity of bacterial communities profiled in wastewater with respect to SARS-CoV-2.**

**A) Principal coordinate analysis of bacterial communities.**

Water samples were categorized into clusters by PCoA using the Bray-Curtis distance metric based on relative abundance of bacterial species. Distance between points indicates degree of dissimilarity in bacterial composition, ranging from zero (samples share the same species abundances) to one (samples contain completely different species abundances). The percent variation explained by each axis is indicated.

**B) Venn diagram representing bacterial communities.**

The number of shared and exclusive bacteria are shown relative to detection genetic mutations associated with SARS-CoV-2 variants of concern, i.e., Alpha (V1) and Beta (V2), along with a sample negative for detection of SARS-CoV-2 (ND).
