# Supplementary material for: Microbiome Analysis for Wastewater Surveillance during COVID-19
Source: mBio. 2022 Jun 21;13(4):e00591-22. doi: 10.1128/mbio.00591-22 (PMC9426581; doi:10.1128/mbio.00591-22)
Supplement: TABLE S1 [file mbio.00591-22-s0006.docx]

**Table S1:** Alpha diversity indices of detected bacteria (DNA) and viruses (RNA).

| Cohort | Sample | Chao1 | Simpson | Shannon |
| --- | --- | --- | --- | --- |
| DNA (bacteria) | 2/11/21 | 1225 | 0.96766398 | 6.641921233 |
|  | 2/17/21 | 1066 | 0.958336218 | 6.371643268 |
|  | 3/10/21 | 1294 | 0.856839186 | 4.996885886 |
|  | 3/23/21 | 1059 | 0.878436055 | 5.090102739 |
|  | 4/15/21 | 1358 | 0.919452894 | 5.887778976 |
|  | 4/21/21 | 1081 | 0.952354203 | 5.840600644 |
| RNA (viruses) | 2/11/21 | 51 | 0.571557453 | 1.729154637 |
|  | 2/17/21 | 34 | 0.559490523 | 1.463624519 |
|  | 3/10/21 | 34 | 0.533781786 | 1.726833027 |
|  | 3/23/21 | 75 | 0.682919217 | 1.78753687 |
|  | 4/15/21 | 29 | 0.694243308 | 1.893986272 |
|  | 4/21/21 | 37 | 0.576365338 | 1.495410593 |
