# Supplementary material for: Microbiome Analysis for Wastewater Surveillance during COVID-19
Source: mBio. 2022 Jun 21;13(4):e00591-22. doi: 10.1128/mbio.00591-22 (PMC9426581; doi:10.1128/mbio.00591-22)
Supplement: FIG S3 [file mbio.00591-22-s0004.docx]

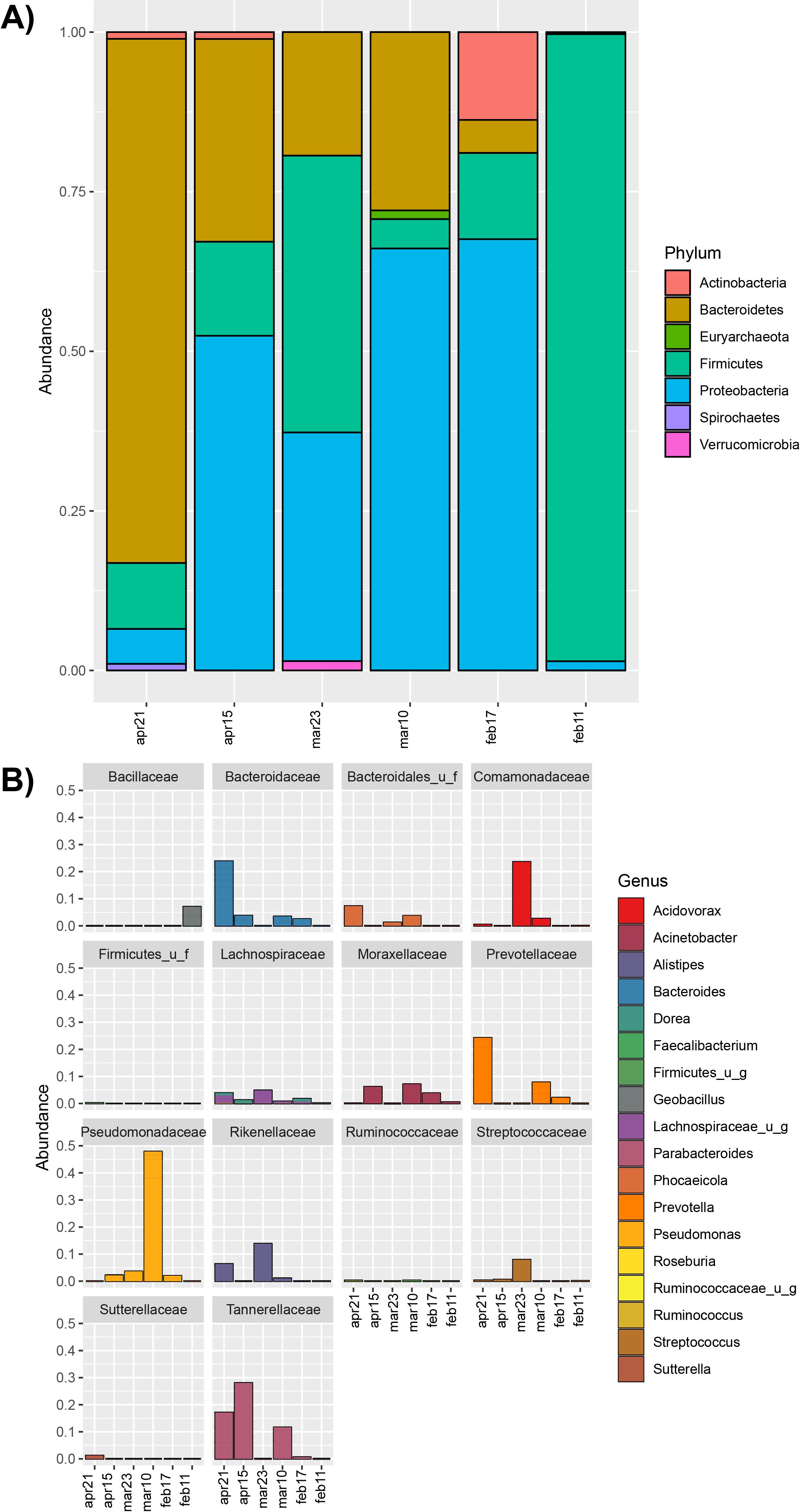


**Figure S3: Microbiome profiles employing RNA metatranscriptomic sequencing.**

**Microbiome profiles employing RNA metatranscriptomic sequencing.**

**A) Stacked bar plot showing relative abundance of detected bacterial phyla.**

**B) Stacked bar plots showing relative abundance of detected bacterial genera.**

Shown are the most abundant genera, grouped by family, detected by both DNA and RNA shotgun sequencing.
