# Supplementary material for: Microbiome Analysis for Wastewater Surveillance during COVID-19
Source: mBio. 2022 Jun 21;13(4):e00591-22. doi: 10.1128/mbio.00591-22 (PMC9426581; doi:10.1128/mbio.00591-22)
Supplement: FIG S4 [file mbio.00591-22-s0005.docx]

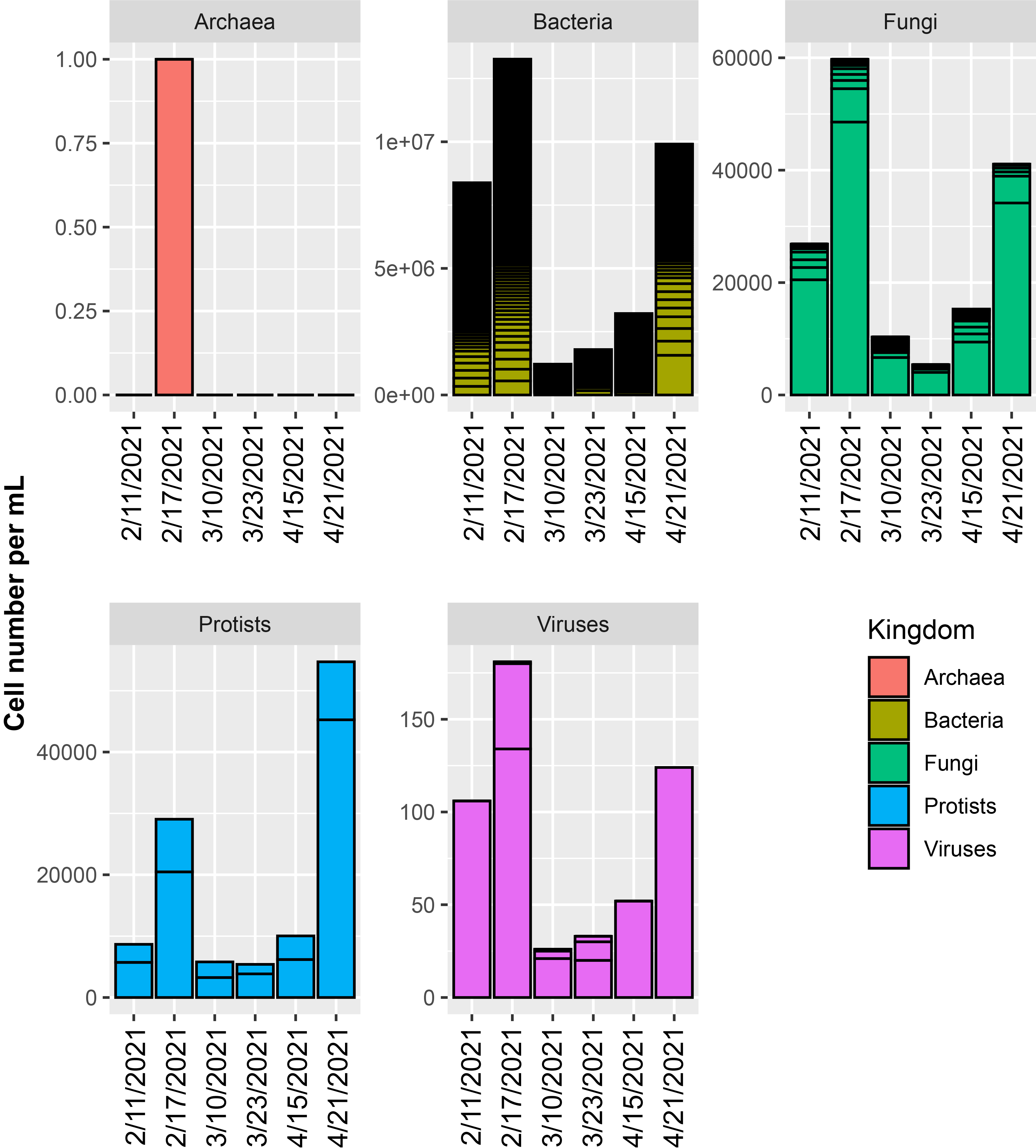


**Figure S4: Stacked bar plots showing quantification (cell number/mL) of detected microbiota (DNA).**

Microbial cell number of each taxon was normalized to the cell number of an *in situ* positive control comprised of *Imtechella halotolerans* (Gram-negative) and *Allobacillus halotolerans* (Gram-positive). Quantification of microbiota are shown as cells per mL and are grouped by kingdom.
