# Supplementary material for: Microbiome Analysis for Wastewater Surveillance during COVID-19
Source: mBio. 2022 Jun 21;13(4):e00591-22. doi: 10.1128/mbio.00591-22 (PMC9426581; doi:10.1128/mbio.00591-22)
Supplement: TEXT S1 [file mbio.00591-22-s0001.docx]

# **Text S1**

**Taxonomic Classification of Whole Metagenome Sequencing Reads.**

Unassembled metagenomic sequencing reads, were analyzed as described previously (1–5), using CosmosID Metagenomics Cloud Application (v.1.0) (6) to achieve multi-kingdom microbiome analysis and profiling of AR associated genes and quantification of the organism RA, defined as the proportion of unique organism-specific k-mers annotated by each database relative to the total number of unique sequencing reads generated for that sample. Briefly, the application utilizes GenBook®, a series of curated reference databases, composed of over 150,000 microbial genomes and gene sequences representing over 15,000 bacterial, 5,000 viral, 250 protozoan, and 1,500 fungal species, as well as over 5,500 AR and virulence-associated genes. The pipeline comprises separate pre-computation and per-sample, computational comparator phases. The pre-computation phase requires a reference microbial database, i.e., GenBook®, as input, and output is a whole genome phylogenetic tree, together with sets of variable-length k-mer fingerprints (biomarkers) that are uniquely identified with distinct branches, nodes, and leaves of the tree. The per-sample, computational phase employs edit distance-scoring techniques, similar in function to BLAST, to compare sequencing reads against the fingerprint sets to provide composition and relative abundance estimates at all branches, nodes, and leaves of the tree. Aggregation statistics are used to maintain overall classification precision. The first comparator phase identifies reads for which there is an exact match with a k-mer uniquely identified in one or a set of reference strains/genes; the second comparator then statistically scores the entire read against the reference to verify that the read is indeed uniquely identified with that set. For each sample, the reads are assigned to the strain/gene with the highest aggregation statistics.

# **References**

1. Brumfield KD, Hasan NA, Leddy MB, Cotruvo J, Rashed SM, Colwell RR, Huq A. 2020. A Comparative Analysis of Drinking Water Employing Metagenomics. PLoS One 15:e0231210.

2. Roy MA, Arnaud JM, Jasmin PM, Hamner S, Hasan NA, Colwell RR, Ford TE. 2018. A Metagenomic Approach to Evaluating Surface Water Quality in Haiti. Int J Environ Res Public Health 15:2211.

3. Lax S, Smith DP, Hampton-marcell J, Owens SM, Shogan BD, Weiss S, Metcalf JL, Ursell LK. 2012. Longitudinal analysis of microbial interaction between humans and the indoor environment. Science (80- ) 12702:1048–1052.

4. Ponnusamy D, Kozlova E V, Sha J, Erova TE, Azar SR, Fitts EC. 2016. Cross-talk among flesh-eating Aeromonas hydrophila strains in mixed infection leading to necrotizing fasciitis. Proc Natl Acad Sci 113:722–727.

5. Connelly S, Fanelli B, Hasan NA, Kaleko M, Colwell RR. 2019. Oral Metallo-Beta-Lactamase Protects the Gut Microbiome From Carbapenem-Mediated Damage and Reduces Propagation of Antibiotic Resistance in Pigs. Front Microbiol 10:1–12.

6. CosmosID. 2019. CosmosID Metagenomics Cloud.
